# Supplementary material for: The effect of enantioselective chiral covalent organic frameworks and cysteine sacrificial donors on photocatalytic hydrogen evolution
Source: Nat Commun. 2022 Oct 1;13:5768. doi: 10.1038/s41467-022-33501-8 (PMC9526734; doi:10.1038/s41467-022-33501-8)
Supplement: Supplementary file 1 — Supplementary Information [file 41467_2022_33501_MOESM1_ESM.pdf]

Supplementary Information

The Effect of Enantioselective Chiral Covalent Organic Frameworks and Cysteine Sacrificial Donors on Photocatalytic Hydrogen Evolution

Weijun Weng[1] and Jia Guo*[1]

[1]*State Key Laboratory of Molecular Engineering of Polymers, Department of Macromolecular Science, Fudan University, Shanghai 200433, China.*

**E-mail: guojia@fudan.edu.cn*

Section I. Materials and Methods

1. Materials

Anhydrous dioxane, anhydrous 1,2-dichlorobenzene (o-DCB), anhydrous *n*-butanol (*n*-BuOH), pyrrolidine, copper acetate monohydrate, L-cysteine, and D-cysteine were purchased from Aladdin Industrial Corporation. *p*-Phenylenediamine (Pa), (*R*)-benzenemethanamine ((*R*)-1-PEA), (*S*)-benzenemethanamine ((*S*)-1-PEA), L-ascorbic acid, sodium ascorbate, D- araboascorbic acid, and sodium D-araboascorbate were purchased from TCI Shanghai. Tetrahydrofuran (THF) was purchased from Shanghai Chemical Regents Company. 1,3,5-Triformylphloroglucinol (Tp), 4,4'-biphenyldiamine (BD), and 4,4''-diaminoterphenyl (TP) were purchased from Jilin Yanshen Technology Co. Ltd. All the chemicals were used without further purification.

2. Characterizations

Powder X-ray diffraction (PXRD) measurements were carried out at room temperature using an X-ray diffraction spectrometer (Bruker D8 Advance, Germany) with Cu Kα radiation (λ = 0.154 nm) at 40 kV and 40 mA. X-ray photoelectron spectroscopy (XPS) and Auger electron spectroscopy were collected on an X-ray photoelectron spectrometer (Thermo Fischer ESCALAB 250Xi, USA) with the monochromatic Al Kα radiation (1486.6 eV) at 14.6kV and 13.5mA. Fourier transform infrared (FT-IR) spectra were tested on a Fourier transformation infrared spectrometer (Thermo Fischer Nicolet 6700, USA) with KBr pellets. UV-vis diffused reflectance spectra were recorded in the solid state by a Lambda 750 spectrometer (Perkin-Elmer, USA) equipped with diffuse reflectance attachment. Circular dichroism spectra were carried out at room temperature using a Chirascan (Applied Photophysics Ltd, UK) and tested with the aqueous dispersion of COFs (100 mg/L). The raw data was plotted without smoothing. N_2 adsorption-desorption isotherms were recorded at 77 K by a TriStar II 3020 volumetric adsorption

analyzer (Micromeritics, USA). Before the test, samples were degassed at 90 °C for 24 h. The pore-size distributions were calculated based on the Nonlocal Density Functional Theory (NLDFT). ^{1}H NMR and ^{13}C NMR spectra were collected on a 400 MHz spectrophotometer (Bruker AVANCE III HD, Switzerland) at 298.15 K. Inductively coupled plasma atomic emission spectroscopy (ICP-AES) was conducted on the iCAP 7400 spectrometer (Thermo Fischer, USA), and the standard curve was renewed before test. The mass spectrometry was collected on a matrix-assisted laser desorption ionization (MALDI) time of flight (TOF)-mass spectrometer (AB SCIEX TOF/TOF 5800 System, USA), using *trans*-2-[3-(4-*tert*-butylphenyl)-2-methyl-2-propenylidene] malononitrile (DCTB) as a matrix. Isothermal titration calorimetry was carried out using the calorimeter (Malvern PeaQ-ITC, UK). The titration of water to water was performed before test. Transmission electron microscopy (TEM) images were recorded using a field 120kV transmission electron microscope (Hitachi HT7800, Japan). High resolution transmission electron microscopy (HR TEM) images were obtained using a field emission transmission electron microscope (FEI Tecnai G2 F20 S-Twin, USA) operating at 200 kV accelerating voltage. The elemental mappings of C, N, O and Cu atoms were collected using the X-Max 80T detector (Oxford Instruments, UK) under the ADF STEM mode. The HADDF-STEM images were obtained using an atomic resolution analytical microscope (JEOL JEM-ARM200F, Japan) equipped with the spherical aberration correction system. The electron paramagnetic resonance (EPR) spectra were recorded using Bruker EMXplus-6/1 (Germany). The deionized water used in measurement was bubbled with N_2 for 30 min to remove oxygen. The electrochemical measurements were recorded on the CHI760E electrochemical workstation (Chenhua, China) with a standard three-electrode system with the photocatalyst-coated ITO as the working electrode, Pt wire as the counter electrode and the Ag/AgCl electrode as a reference electrode. The electrolytes were bubbled with Ar for 1h before the measurement. The lifetimes were examined with a 450-nm diode laser using Time-Correlated Single Photon Counting (TCSPC) technique and calculated by fitting with first-order

exponential curve.

3. Methods

3.1 Synthesis of Tp-based COFs

The synthesis of Tp-based COFs was carried out using the reported solvothermal method.[1] 0.08 mmol 1,3,5-triformylphloroglucinol (Tp) and 0.12 mmol diamine monomers were charged into a Pyrex tube (10 cm×1cm) and dispersed in 1 mL solvent (dioxane for TpPa-COF and a mixture of DCB and n-BuOH (v/v, 9/1) for TpBD-COF and TpTP-COF). Then 0.02 mL pyrrolidine was added and the mixture was dispersed by sonification. After three freeze-pump-thaw cycles, the tube was sealed off and kept in the oven at 120°C for 3 days. Afterward, the precipitate was filtered, washed with THF (3×10 mL), extracted by Soxhlet with THF for 24 h, and dried under vacuum at 40°C for 24 h. Finally, the product was obtained with a 75-85% yield.

3.2 Synthesis of TpPa amorphous polymer

33.6 mg Tp and 25.9 mg Pa were dissolved in 30 mL THF by sonication for 10 min. The mixture was stirred at room temperature for 12 h. The product was collected by filtration and washed by Soxhlet extraction with THF for 24 h. The resulting polymer was dried under vacuum at 40°C for 48 h to afford the red powder with a 85% yield.

3.3 Synthesis of TpPa-Cu(I)-COF and TpPa(Δ)-Cu(I)-COF

L-cysteine (0.1 mol/L) was mixed with the aqueous dispersions of TpPa-Cu(II)-COF or TpPa(Δ)-Cu(II)-COF (10 mg, 100 mL) and the solution was degassed in the dark. After stirring for 1h, the product was collected by filtration, washed with water several times, and dried under vacuum overnight. The product was preserved in a dark brown bottle shielded from light and protected in N_2 atmosphere. If no

otherwise specified, TpPa-Cu(I) and TpPa(Δ)-Cu(I) were prepared in the same way.

3.4 Synthesis of model compounds

TpPa model

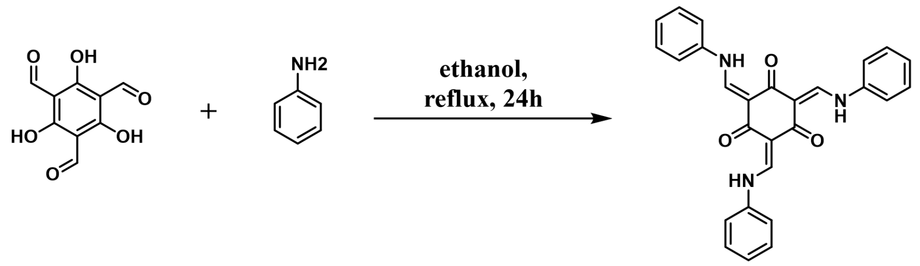

Triformylphloroglucinol (0.081 g, 0.4 mmol) and aniline (0.250 g, 2.7 mmol) were dissolved in ethanol (35 mL). The reaction proceeded under the reflux condition for 24 h. After cooling to room temperature, the precipitate was filtered and washed with anhydrous ethanol several times. The yellowish product was obtained after drying under vacuum for 24 h (0.156 g, 0.36 mmol, 90%). ^{1}H NMR (400 MHz, $CDCl_3$): δ 12.9-13.5 (m, NH), 8.7-8.9 (d, CH), and 7.15-7.5 (m, partially overlapping with solvent peak, Ph). ^{13}C NMR (400 MHz, $CDCl_3$): δ 185.54, 149.33, 139.10, 129.90, 125.68, 117.73, 106.73. Maldi-TOF MS: calculated $[M]^+$: 435.1 Da, found: 434.9.

TpPa-Cu(II) model

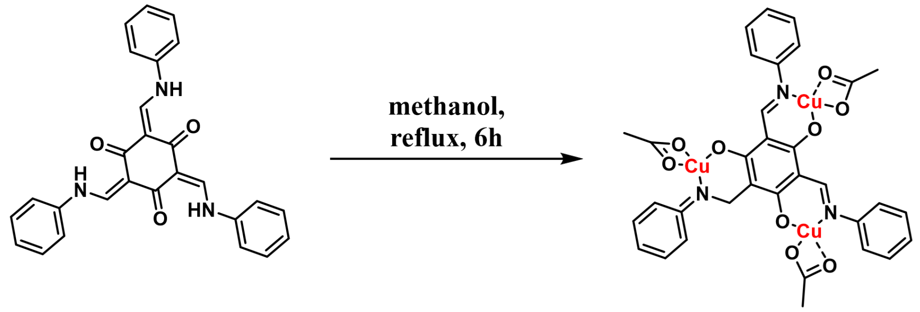

60 mg $Cu(OAc)\cdot H_2O$ (0.3 mmol) was dissolved in 10 mL methanol and 43.5 mg 2,4,6-tris((phenylamino)methylene)cyclohexane-1,3,5-trione (0.1 mmol) was added with another 10 mL of methanol. The mixture was stirred under reflux for 6 h. The product was collected by filtration, washed with methanol several times, and

dried under vacuum at 40°C for 24h. The coordinated Cu content: calculated: 23.79 wt%, found: 24.40 wt% (measure by ICP-AES). Maldi-TOF MS: calculated [M+H-$Cu(OAc)_3$]$^+$: 560.0 Da, found: 562.6. Elemental analysis: calculated: N, 5.24; C, 49.47; H, 3.52, found: N, 5.66; C, 51.92; H, 3.37.

3.5 X-ray absorption fine structure (XAFS) analyses

Cu K-edge analysis was performed with Si(111) crystal monochromators at the BL11B beamlines at the Shanghai Synchrotron Radiation Facility (SSRF) (Shanghai, China). Before the analysis at the beamline, samples were pressed into thin sheets with 1 cm in diameter and sealed using Kapton tape film. The XAFS spectra were recorded at room temperature, using a 4-channel Silicon Drift Detector (SDD) Bruker 5040. Cu K-edge extended X-ray absorption fine structure (EXAFS) spectra were recorded in the transmission mode. The spectra were analyzed by the software codes Athena and Artemis.[2] The Morlet wavelet transformation was performed by codes hamaFortran.[3]

In the fitting process, coordination number (N), amplitude attenuation factor (S_0^2), interatomic distance (R), Debye-Waller factor (σ^2) and edge energy shift (ΔE_0) were all taken into consideration. The effective scattering amplitude, effective scattering phase shift and mean free path were calculated by FEFF6. The amplitude attenuation factor (S_0^2) was determined to be 0.875 by fitting the spectra of Cu foil with a constrained coordination number 12. The fluctuation of S_0^2 was neglectable as the data were collected simultaneously. The R factor indicated the goodness of the fit.

3.6 Photoelectrochemical/electrochemical measurements

The photocurrent response profiles, electrochemical impedance spectra (EIS), Mott-Schottky plots and cyclic voltammogram were recorded on the CHI 760E electrochemical workstation with a standard three-electrode system, using the Ar-

purged 0.2M Na_2SO_4 as the electrolyte, sample-coated ITO as the working electrode, Pt wire as the counter electrode and Ag/AgCl electrode as the reference electrode. The ethanol dispersion of samples was drop-cast onto the ITO with the mass loading of 0.1 mg/cm^2. The 300 W Xe lamp (λ ≥ 420 nm) with a power density of 100 mW·cm^{-2} was used as the light source.

The polarization curves were collected using linear sweep voltammetry at the scan rate of 2 mV s^{-1}. 0.5M H_2SO_4 (pH=0) aqueous solution was applied as the electrolyte after bubbling Ar for 1 h. Pt wire was the counter electrode and the Ag/AgCl electrode was the reference electrode. The sample (1 mg) was dispersed in 1 mL ethanol and mixed with 50 μL 5wt% Nafion solution. Then 10μL dispersion was drop-cast onto the polished glass carbon electrode to form the working electrode.

The conversion between potentials *vs.* Ag/AgCl, those *vs.* RHE and those *vs.* NHE follows the equation below:

$$\mathrm{E}(vs.\,\mathrm{RHE}) = \mathrm{E}(vs.\,\mathrm{Ag/AgCl}) + \mathrm{E}_{Ag/AgCl} + 0.059\mathrm{V} \times \mathrm{pH} \qquad (1)$$

$$\mathrm{E}(vs.\,\mathrm{NHE}) = \mathrm{E}(vs.\,\mathrm{Ag/AgCl}) + \mathrm{E}_{Ag/AgCl} \qquad (2)$$

($E_{Ag/AgCl}$=0.1967 V *vs.* NHE at 25 °C)

3.7 AQE calculation

The apparent quantum efficiency (AQE) was measured under the irradiation of a Xe lamp equipped with different bandpass filters (λ_0 ± 20 nm), followed by the calculation using the following equation:

$$\eta_{\mathrm{AQE}} = \frac{N_e}{N_p} \times 100\% = \frac{2\times M \times N_A}{\frac{E_{total}}{E_{photon}}} \times 100\% = \frac{2 \times M \times N_A \times \hbar \times c}{S \times P \times t \times \lambda} \times 100\% \qquad (3)$$

Where, M is the amount of H_2 molecules (mol), N_A is the Avogadro constant (6.022 ×10^{23} mol^{-1}), $\hbar$ is the Planck constant (6.626 × 10^{-34} J s), c is the light speed (3 ×10^8 m s^{-1}), S is the irradiation area (cm^2), P is the irradiation intensity (W cm^{-2}), t is the

photoreaction time (s), λ is the wavelength of the monochromatic light (m).

4. Calculations

4.1 Calculation of Models

The DFT calculation of model structures was performed by Gaussian 16 software package.[4] The models derived from the reported crystal structure were fully optimized at the PBE0-D3BJ/ma-def2-SVP level.[5,6] The implicit solvation model (IEF-PCM) of water was applied in calculation. The vibrational frequency calculation was carried out at the same level. The single point calculations of vertical excitation were performed using the time-dependent DFT method at the level of TD-PBE0-D3BJ/ma-def2-SVP without further relaxation.

The electrostatic potential and electron-hole analysis were processed using Multiwfn 3.8[7] and visualized by VMD 1.9.3 package.[8] The photo-induced charge transfer was quantified based on the interfragment charge transfer method.

The hydrogen binding energy (HBE)[9] was calculated from the follow equation:

$$\Delta G_{\mathrm{HBE}} = G_{*\mathrm{H}} - G_{*} - \frac{1}{2} G_{\mathrm{H}_2} \quad (4)$$

Where *H presents for the structure absorbed H, * is the pristine model, and the 1/2 Gibbs energy of H_2 was applied to approximate the energy of the H atom and single electron.

In the calculation of the electronic circular dichroism, C, N, O atoms were directly cut from the crystal structure without further relaxation, while H atoms were optimized at the level of PBE0-D3BJ/ def2-SVP. Then the vertical excitation calculations were performed at TD-PBE0-D3BJ/ def2-SVP level for at least 30 states.

4.2 Calculation in periodic boundary conditions

The calculations based on the first principle were performed using the CP2K package[10] and visualized by VESTA.[11] In the geometry optimization, the mixed Gaussian and plane waves (GPW) method was applied using the Perdew-Burke-Ernzerhof (PBE) exchange-correlation functional with the DFT-D3(BJ) dispersion correction, based on the GTH pseudopotentials with DZVP-MOLOPT-SR gaussian and plane wave basis set with the cutoff of 600 Ry mapped on a 4-level multigrid. The relative cutoff was set to 50 Ry with the progression factor of 3. The efficient orbital transformation (OT) method and Quickstep code were used to accelerate the calculation. All structures were optimized by the Broyden-Fletcher-Goldfarb-Shanno (BFGS) minimizer, and the SCF convergence criterion was set to 5×10^{-7} to increase the accuracy of density matrix. The frequency analysis was performed at the same level.

Derived from the reported crystal structure of TpPa-COF,[12] the corresponding structures of parallel stacking and antiparallel stacking were built and relaxed. The optimization of H-adsorbed structures was carried out, while the carbon backbone of the parent TpPa-COF was constrained.

The Gibbs free energies (at T K) of different structures were calculated as:

$$G\,(\mathrm{T}) = E_{\mathrm{DFT}} + \Delta G_{\mathrm{cor}}\,(\mathrm{T}) \tag{5}$$

where $\Delta G_{\mathrm{cor}}\,(\mathrm{T}) = \Delta E_{\mathrm{ZPE}} - T\Delta S + \Delta U(\mathrm{T})$ was calculated at 298.15K by processing the frequency analysis results with Shermo 2.3 code[13].

The relative Gibbs free energy for each step of Volmer-Tafel proton reduction was computed as:

$$G_{*+2\mathrm{H}^++2\mathrm{e}^-} = G_* + 2\times \mathrm{G}_{\mathrm{H}^++\mathrm{e}^-} \tag{6}$$

$$\Delta G_{*\mathrm{H}+\mathrm{H}^++\mathrm{e}^-} = \mathrm{G}_{*\mathrm{H}} + \mathrm{G}_{\mathrm{H}^++\mathrm{e}^-} - \mathrm{G}_{*+2\mathrm{H}^++2\mathrm{e}^-} \tag{7}$$

$$\Delta G_{*H+*H} = G_{*H+*H} - G_{*+2H^{+}+2e^{-}} \quad (8)$$

$$\Delta G_{*+H_2} = G_{*+H_2} - G_{*+2H^{+}+2e^{-}} \quad (9)$$

where *H and * present for the structure with and without adsorption of the H atom in the vacuum. Based on the computational hydrogen electrode (CHE) model,[14] the Gibbs free energy of (H^{+}+ e^{-}) was equal to that of half a H_2 molecule.

Section II. Supplementary Figures and Tables

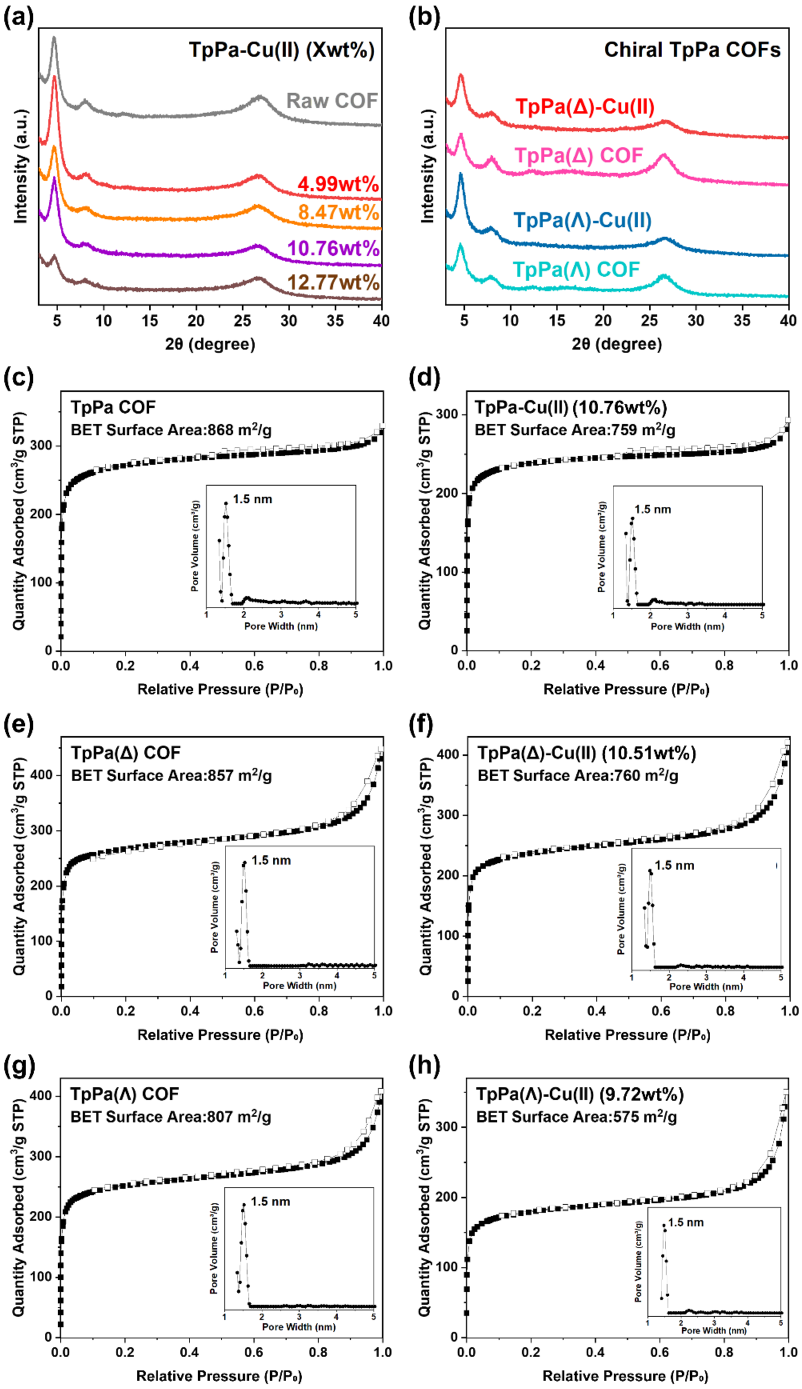

Supplementary Figure 1. PXRD patterns, N_2 sorption isotherms and Pore-size Distribution.

PXRD patterns of (a) the TpPa-Cu(II)-COF(Xwt%) (X=4.99, 8.47, 10.76, 12.77) and (b) the chiral TpPa-COFs and TpPa-Cu(II)-COFs. N_2 sorption isotherms of (c) TpPa-COF, (d) TpPa-Cu(II)-COF(10.76wt%), (e, g) the chiral TpPa-COFs and (f, h) the chiral TpPa-Cu(II)-COFs. Insets in (c-h) are the pore-size distributions.

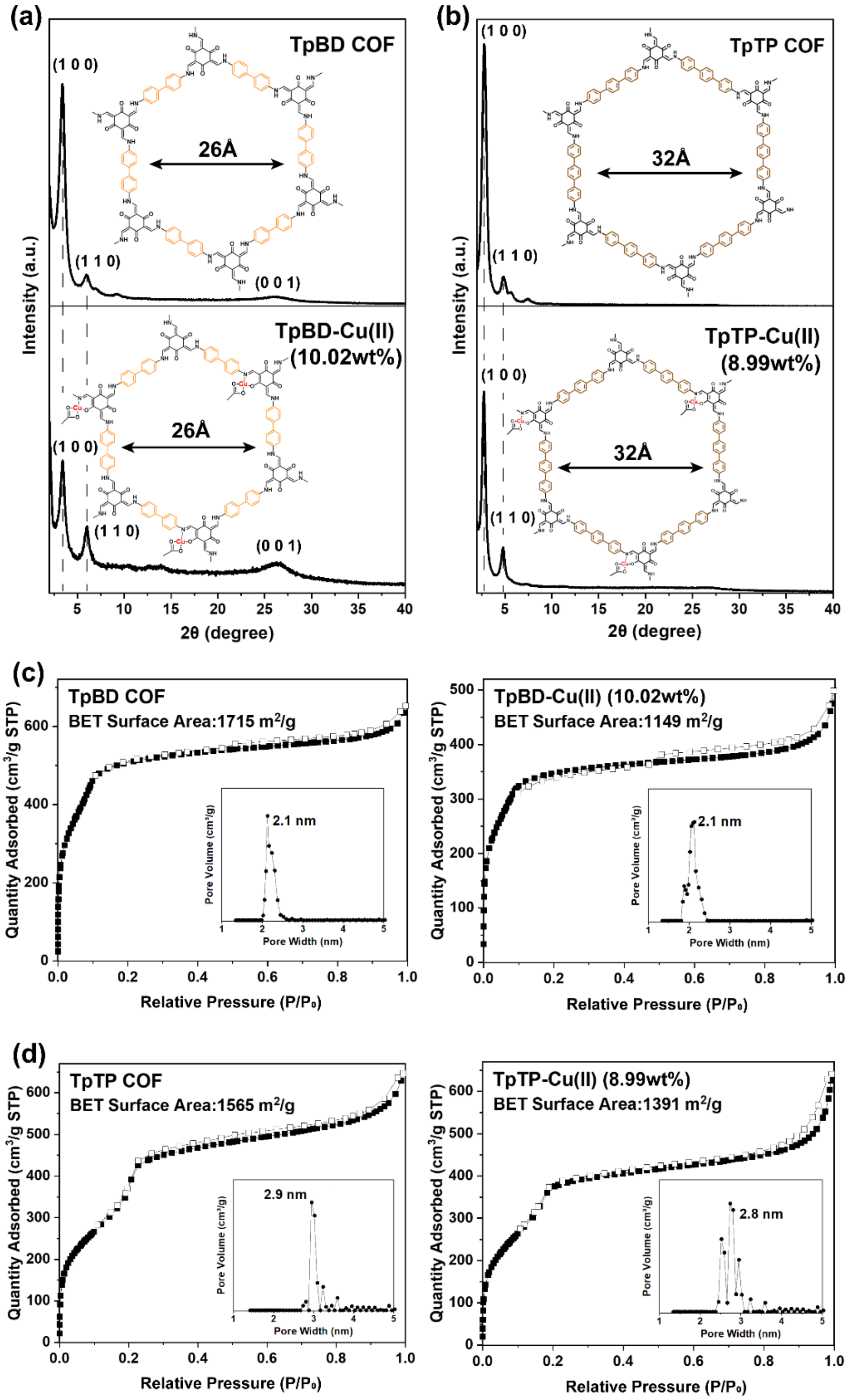

Supplementary Figure 2. PXRD patterns, N_2 sorption isotherms and Pore-size Distribution.

(a,b) PXRD patterns and (c,d) N_2 sorption isotherms of TpBD-COF, TpBD-Cu(II)-CO(10.02wt%), TpTP-COF, and TpTP-Cu(II)-COF(8.99wt%). Insets in (c) and (d) are the pore-size distributions.

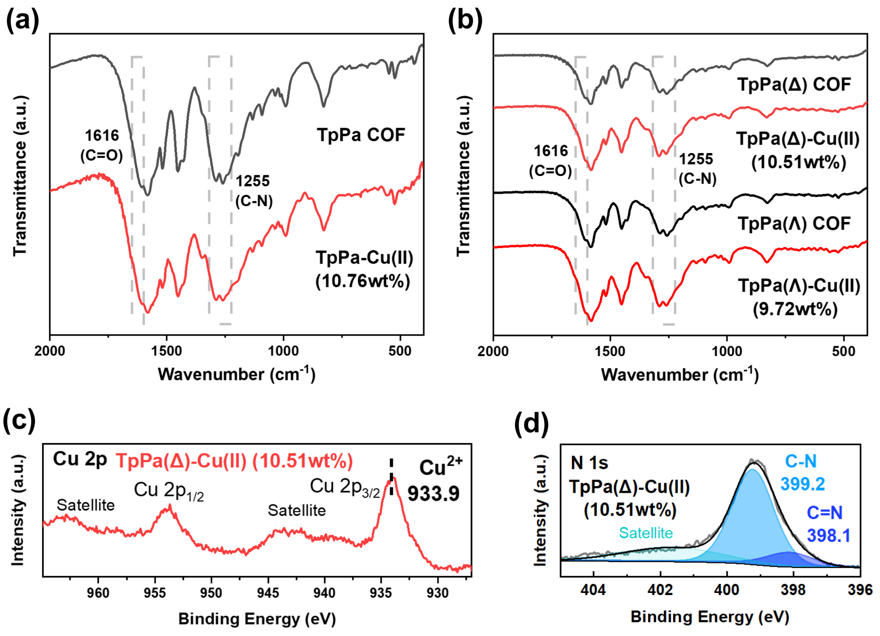

Supplementary Figure 3. FT IR and XPS spectra.

FT IR spectra of (a) the TpPa-COF and TpPa-Cu(II)-COF(10.76wt%) and (b) the chiral TpPa-COFs and TpPa-Cu(II)-COFs. (c) Cu 2*p* XPS spectrum of the TpPa(Δ)-Cu(II)-COF(10.51wt%). (d) N 1*s* XPS spectrum of the TpPa(Δ)-Cu(II)-COF(10.51wt%).

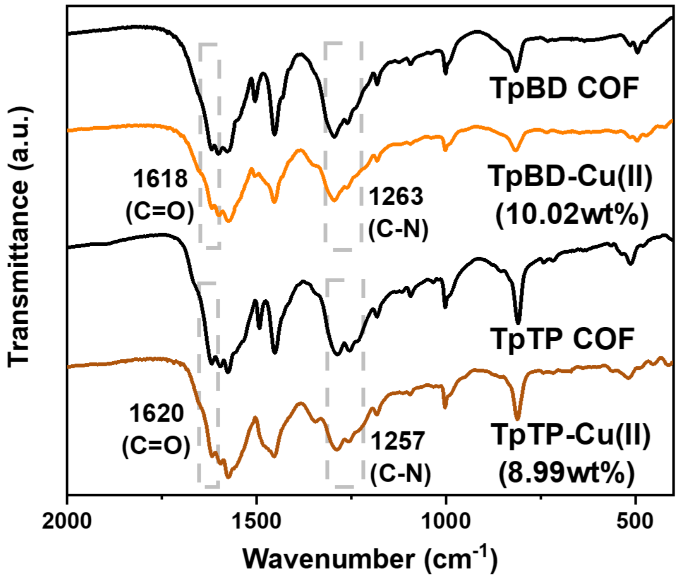

Supplementary Figure 4. FT IR spectra.

FT IR spectra of TpBD-COF, TpBD-Cu(II)-COF(10.02wt%), TpTP-COF, and TpTP-Cu(II)-COF(8.99wt%).

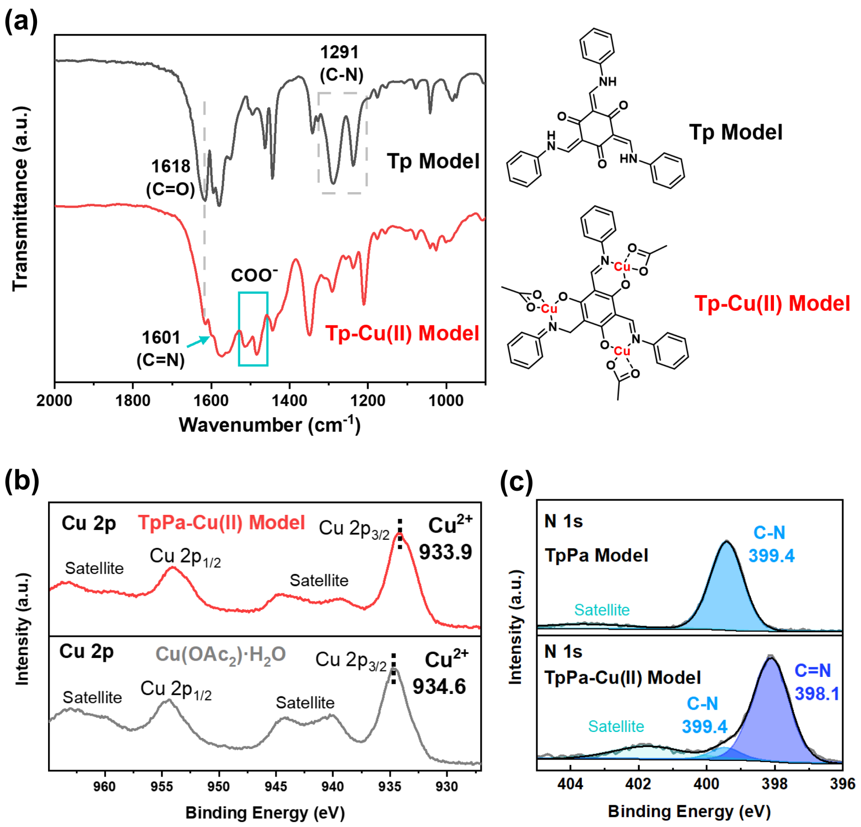

Supplementary Figure 5. FT IR and XPS spectra.

(a) FT IR spectra of the TpPa model and TpPa-Cu(II) models. (b) Cu 2*p* XPS spectra of the TpPa-Cu(II) model and $Cu(OAc)_2{\bullet}H_2O$. (c) N 1*s* XPS spectra of the TpPa model and TpPa-Cu(II) model.

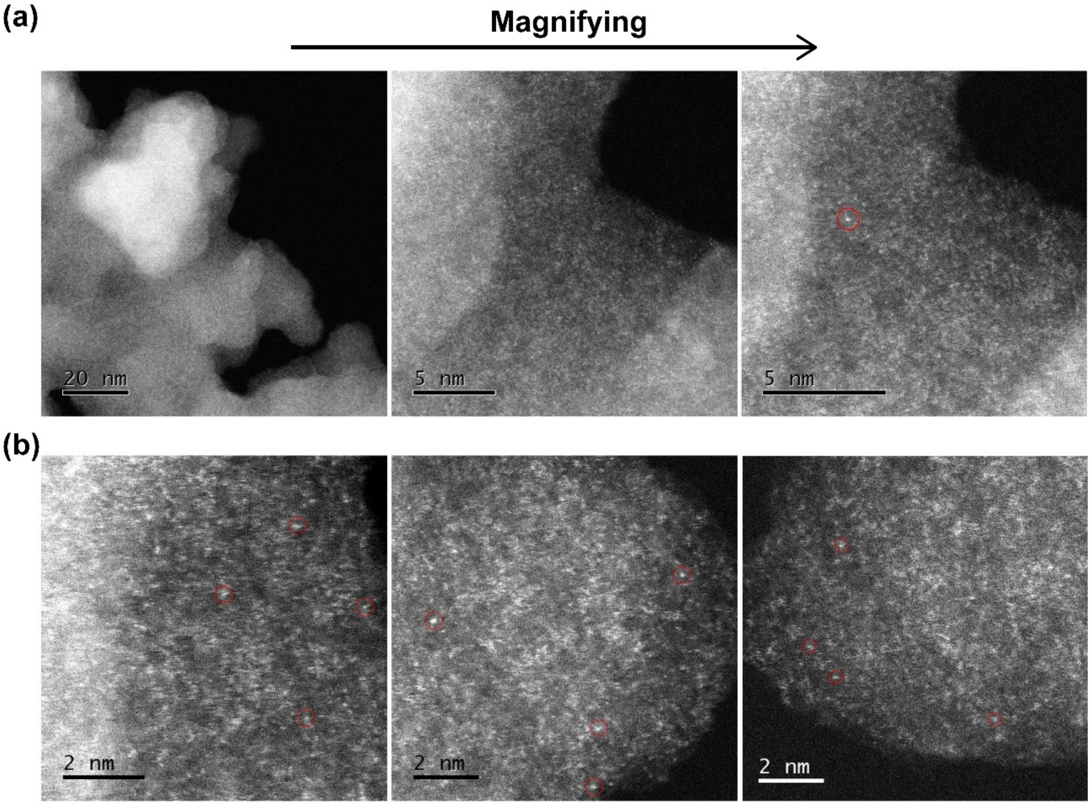

Supplementary Figure 6. HAADF-STEM images of TpPa-Cu(II)-COF.
HAADF-STEM images of the TpPa-Cu(II)-COF(10.76wt%) showing the metal-atom distribution (a) in the gradually magnified area and (b) in the different areas.

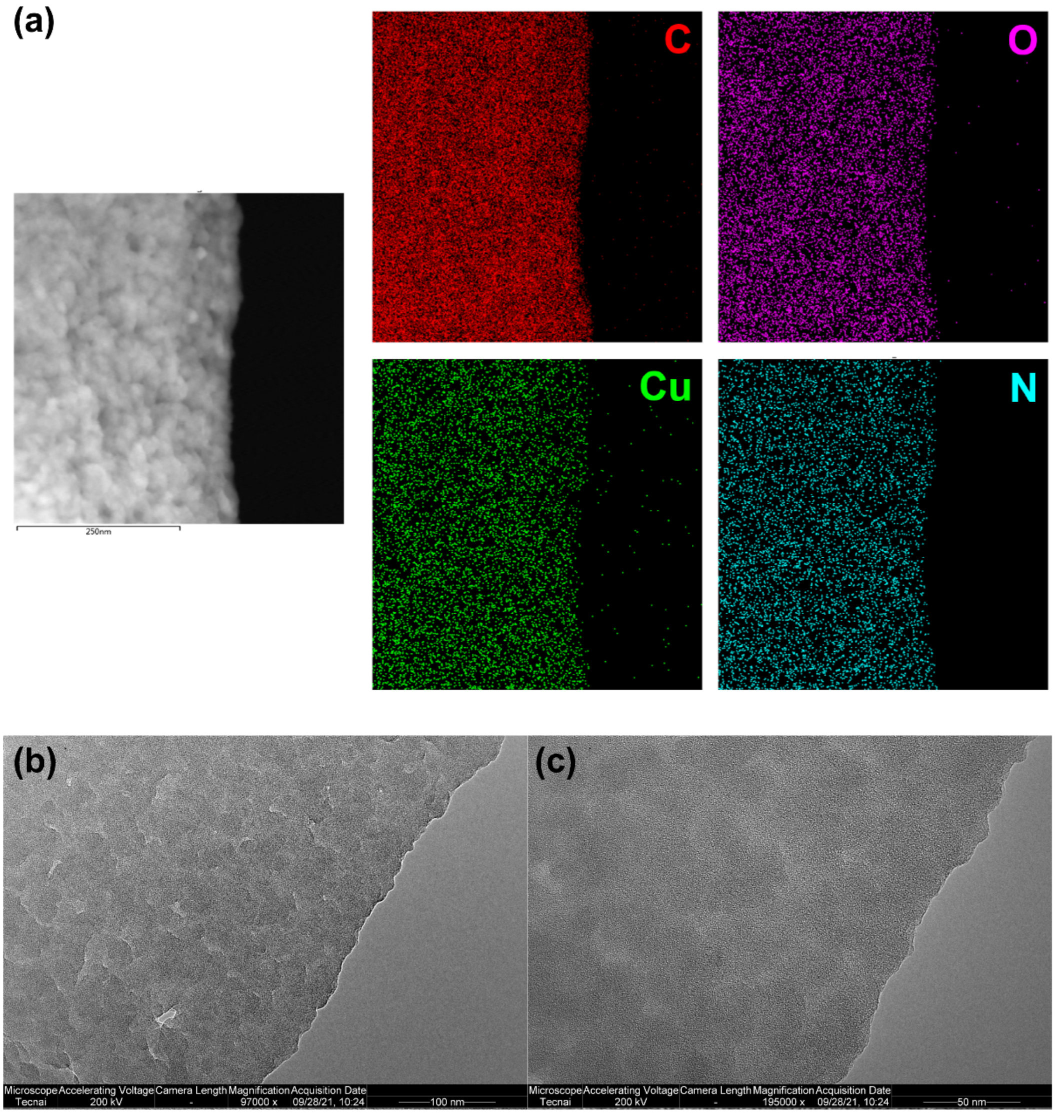

Supplementary Figure 7. HRTEM-DES elemental mapping and HR TEM images.

(a) HRTEM-EDS elemental mapping for the TpPa-Cu(II)-COF(10.76wt%). (b,c) HR TEM images of the TpPa-Cu(II)-COF(10.76wt%) with the different magnification.

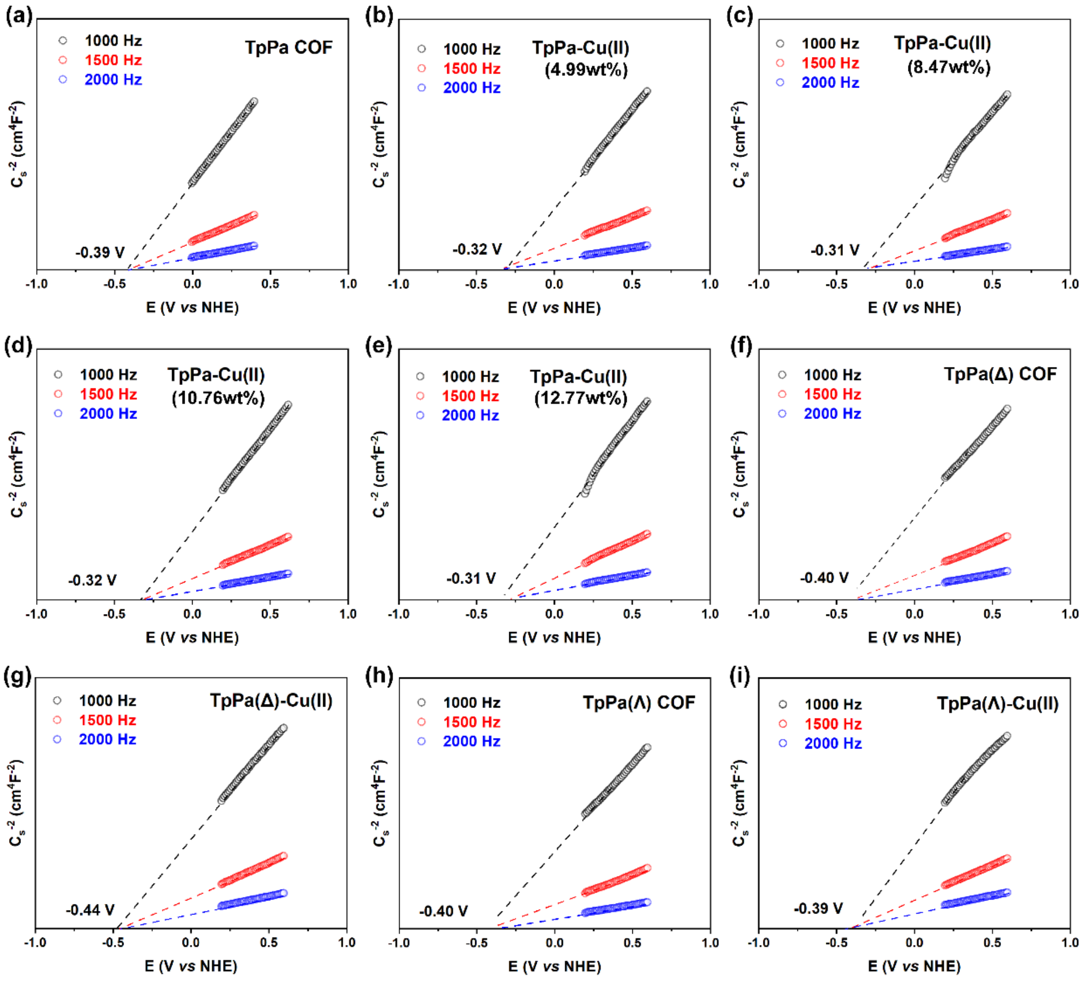

Supplementary Figure 8. Mott-Schottky curves.

Mott-Schottky curves of (a) the TpPa COF, (b-e) TpPa-Cu(II) COFs with 4.99wt%, 8.47wt%, 10.76wt%, 12.77wt% of the coordinated Cu(II), respectively, (f,h) chiral TpPa COFs, and (g, i) chiral TpPa-Cu(II) COFs.

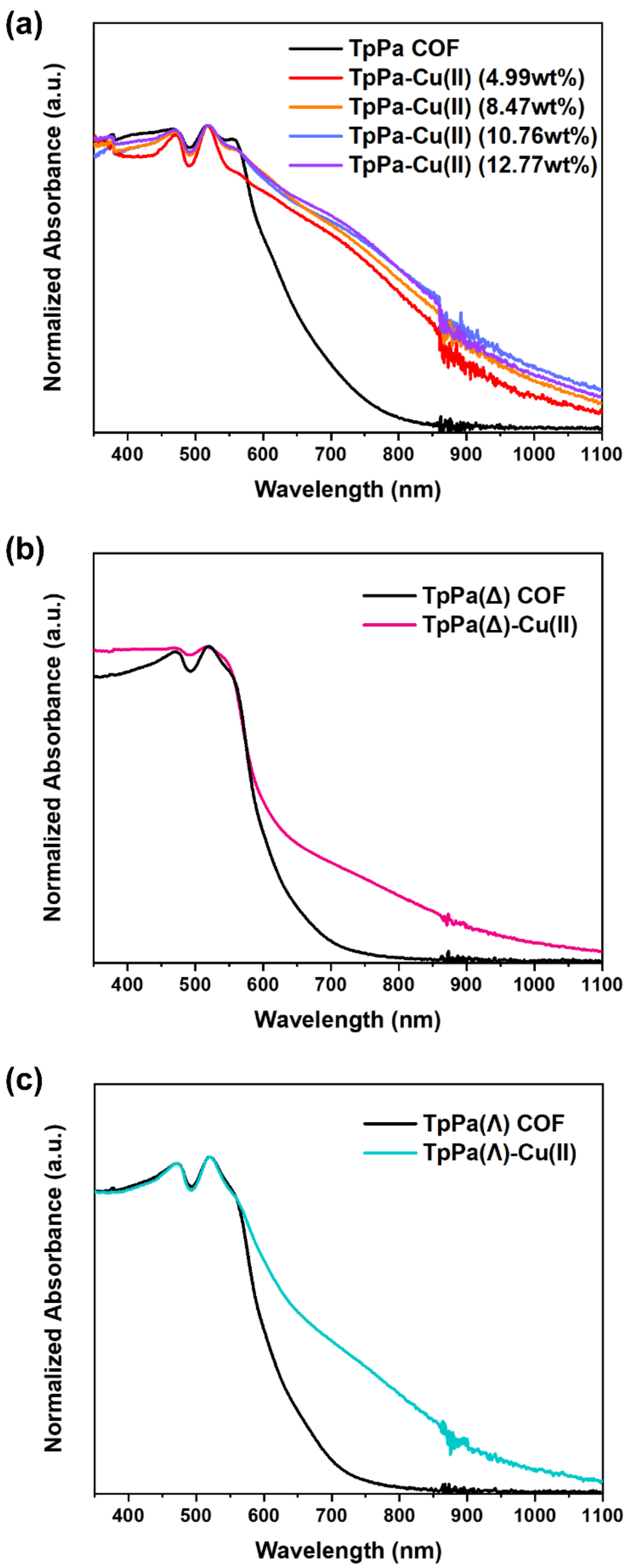

Supplementary Figure 9. Solid-state UV-vis diffuse reflection spectra.
Solid-state UV-vis diffuse reflection spectra of (a) the TpPa-Cu(II) COFs containing 4.99wt%, 8.47wt%, 10.76wt%, 12.77wt% of the coordinated Cu(II), respectively, and (b, c) the chiral TpPa-COF and TpPa-Cu(II)-COF.

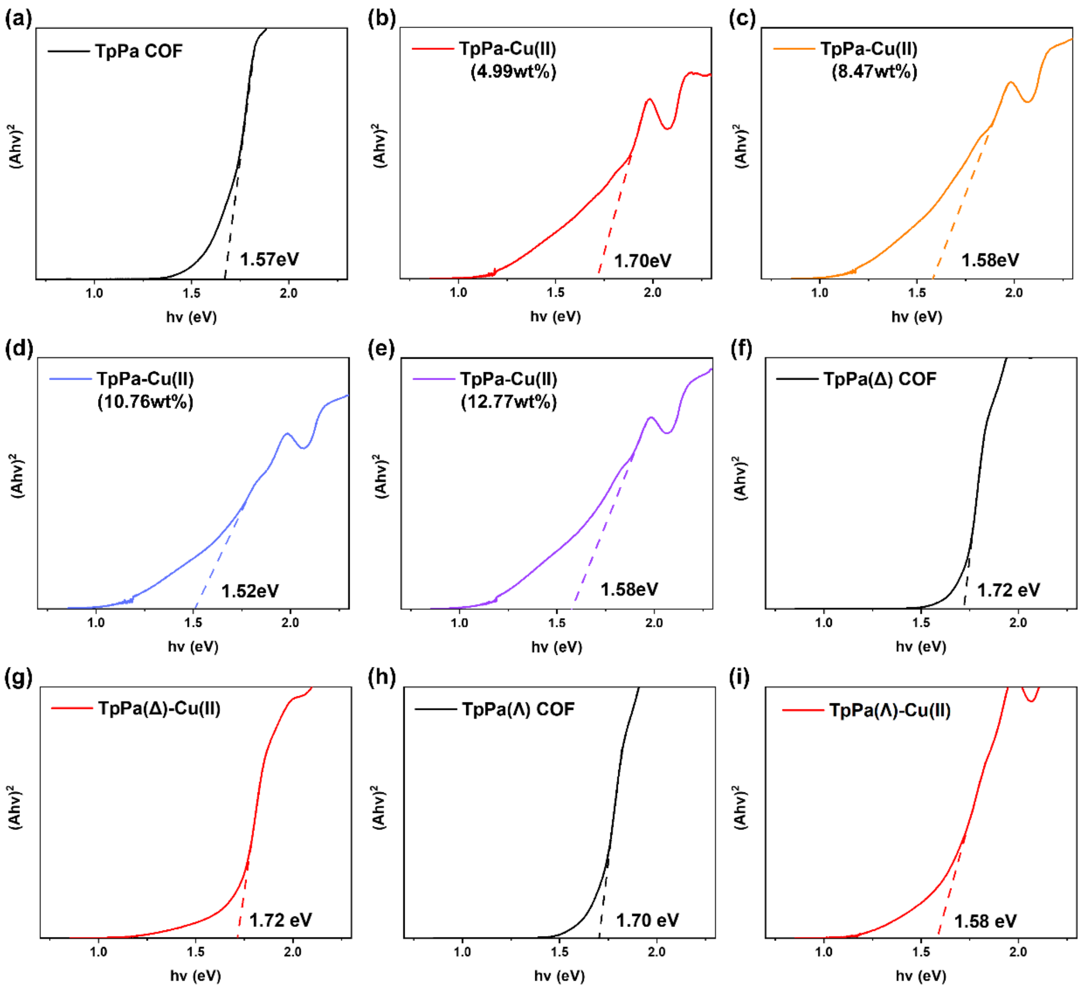

Supplementary Figure 10. Tauc plots.

Tauc plots of (a-e) the TpPa-Cu(II) COFs containing 4.99wt%, 8.47wt%, 10.76wt%, and 12.77wt% of the coordinated Cu(II), respectively, and (f-i) the chiral TpPa-COF and TpPa-Cu(II)-COF.

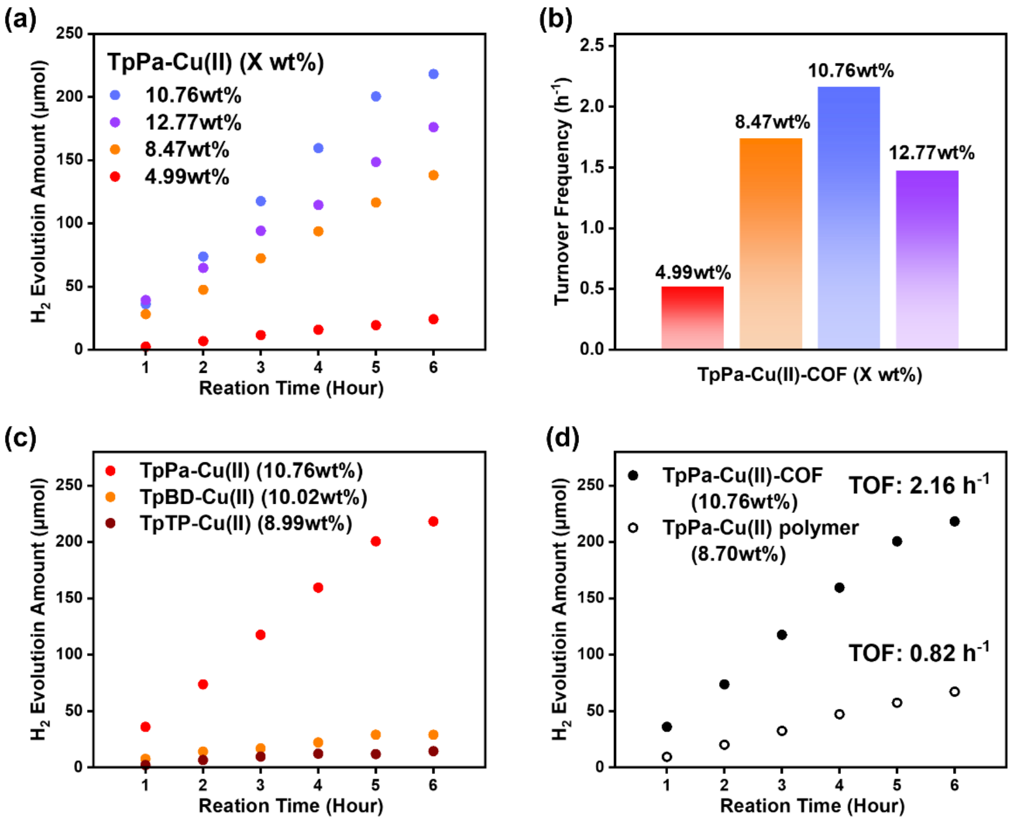

Supplementary Figure 11. Visible photocatalytic H_2 evolution amount *vs.* reaction time and sacrificial oxidation turnover frequency.

(a,c,d) Time courses for photocatalytic H_2 evolution under visible irradiation for (a) the TpPa-Cu(II) COF(Xwt%) (X = 4.99, 8.47, 10.76, 12.77), (c) the TpPa-Cu(II)-COF(10.76wt%), TpBD-Cu(II)-COF(10.02wt%) and TpTP-Cu(II)-COF(8.99wt%), and (d) the TpPa-Cu(II)-COF(10.76wt%) and TpPa-Cu(II)(8.70wt%) amorphous polymer. (b) Histogram of the sacrificial oxidation turnover frequency (TOF_{ox}) for the TpPa-Cu(II)-COF(Xwt%) (X = 4.99, 8.47, 10.76, 12.77) upon exposure to 6-h irradiation.

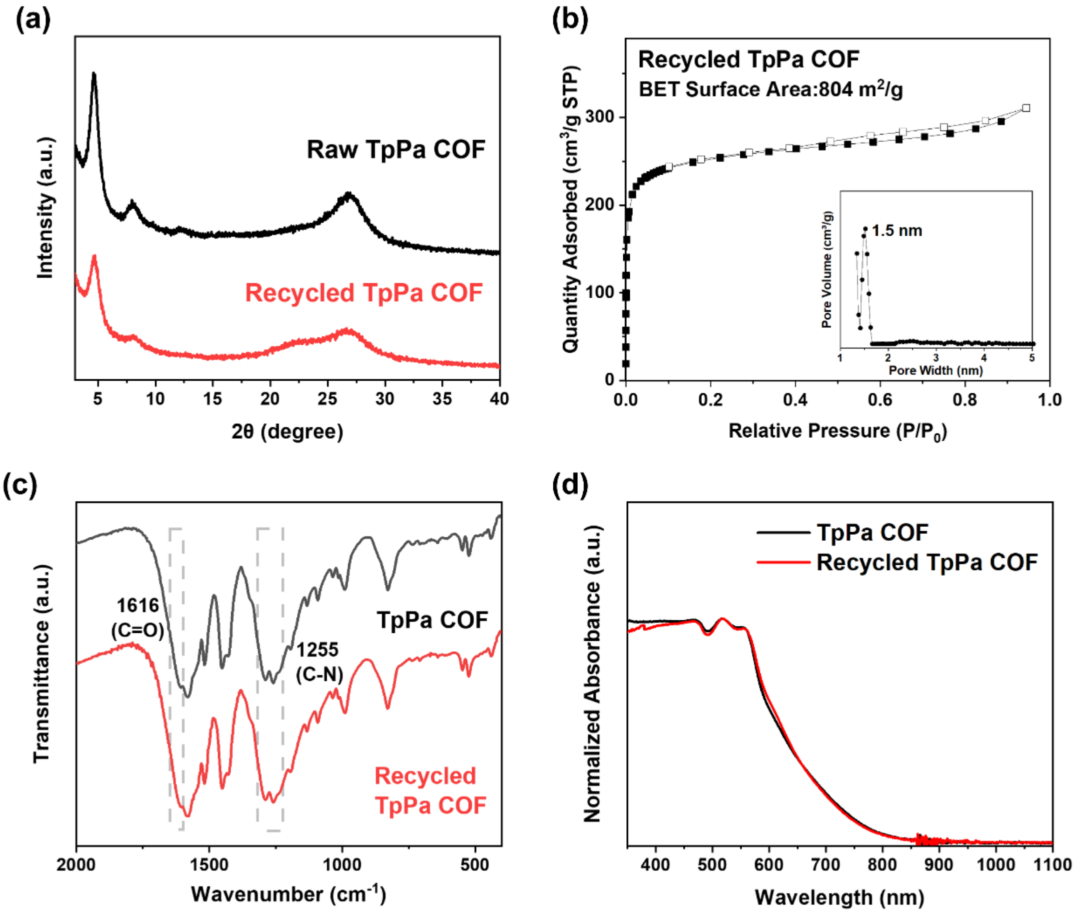

Supplementary Figure 12. PXRD patterns, N_2 sorption isotherms, pore-size distribution, FT IR spectra and UV-vis DRS spectra.

(a) PXRD pattern, (b) N_2 adsorption isotherm (Inset: pore-size distribution), (c) FT IR spectra, and (d) UV-vis DRS spectra of the TpPa-COF before and after 12-h irradiation. The recycled photocatalyst was subjected to acid treatment to remove cystine. The TpPa-Cu(II)-COF complex was decomposed simultaneously. Thus, the TpPa-COF without Cu(II) was used to compare with the recycled COF.

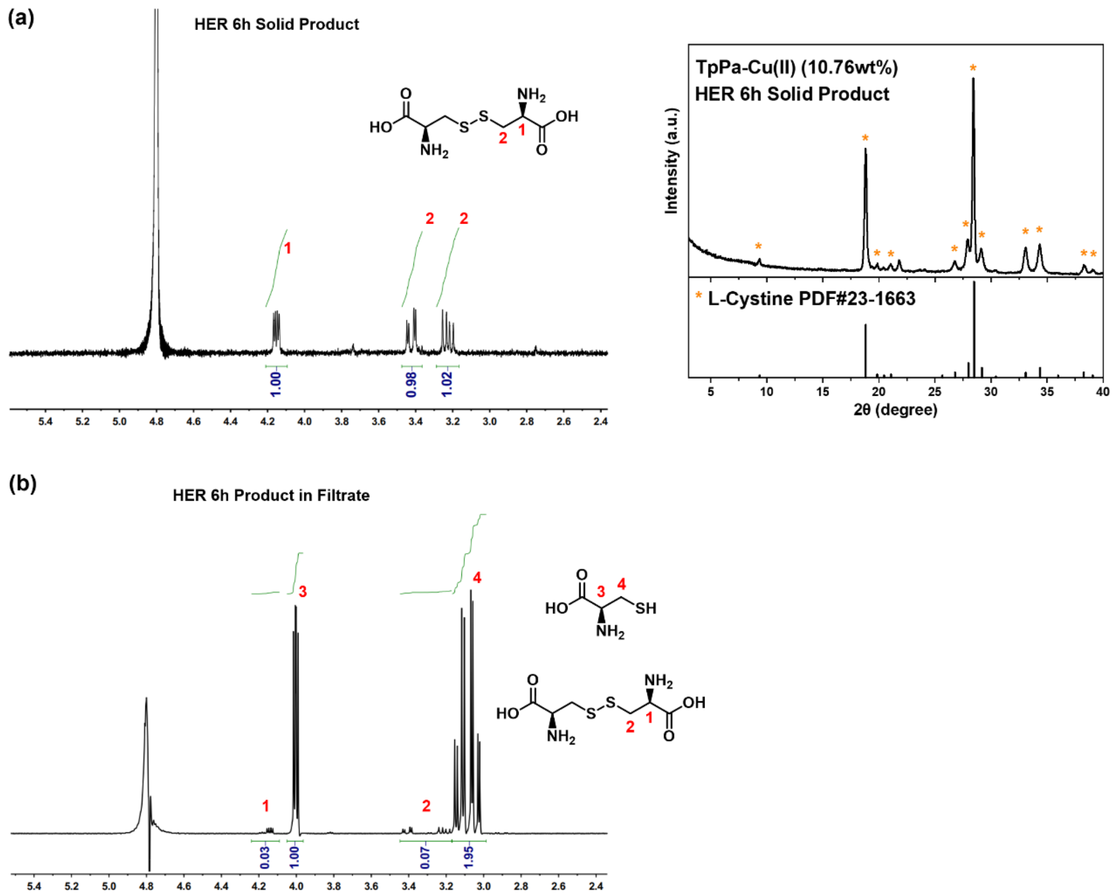

Supplementary Figure 13. ^{1}H NMR spectra and PXRD patterns.

(a) ^{1}H NMR spectrum and PXRD pattern of the precipitate obtained after 6-h photocatalytic reaction. (b) ^{1}H NMR spectrum of the filtrate after 6-h photocatalysis.

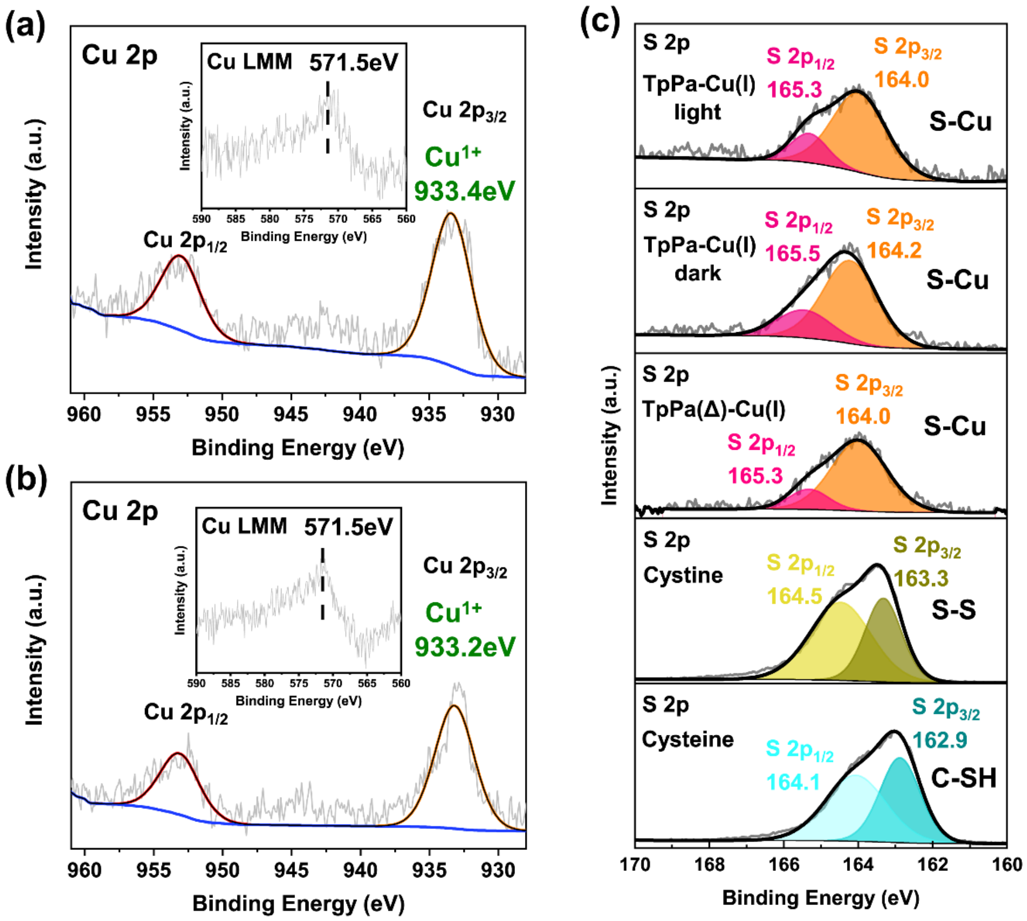

Supplementary Figure 14. Cu 2*p* and S 2*p* XPS spectra.

Cu 2*p* XPS spectra of (a) the TpPa-Cu(I)-COF and (b) the TpPa(Δ)-Cu(I)-COF. (c) S 2*p* XPS spectra of cystine, cysteine, TpPa-Cu(I)-COF(light), TpPa-Cu(I)-COF(dark), and TpPa(Δ)-Cu(I)-COF, respectively. Insets in (a) and (b) are the Cu LMM Auger spectra. TpPa-Cu(I)-COF(light) was recycled after 1-h photocatalysis in the presence of cysteine. TpPa-Cu(I)-COF(dark) and TpPa(Δ)-Cu(I)-COF were recycled after the dark reaction with cysteine.

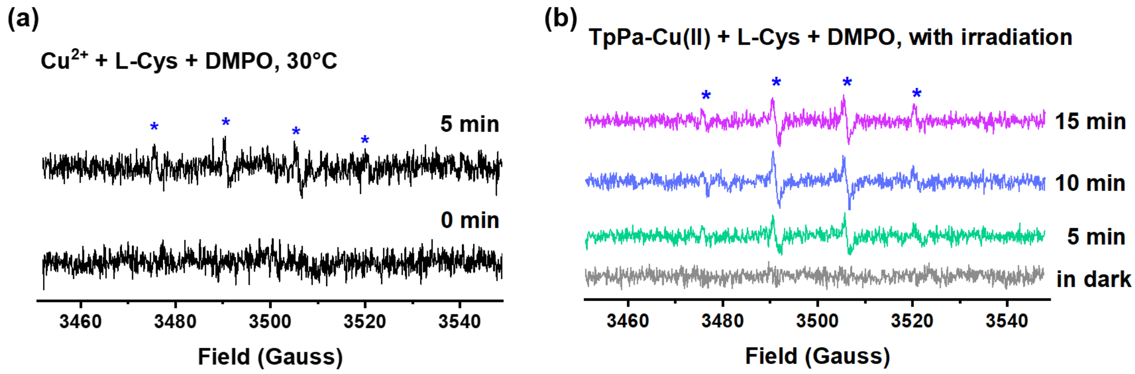

Supplementary Figure 15. Electron paramagnetic resonance (EPR) spectra. (a) EPR spectra of a mixture of L-cysteine (0.15 mol/L), Cu^{2+} aqueous solution (7.5 mmol/L) and DMPO (0.5vol%) recorded at beginning and after 5min at 30°C (CysS·, marked with *), respectively. (b) EPR spectra of a mixture of TpPa-Cu(II)-COF (100 mg/L), L-cysteine (0.1 mol/L) and DMPO (0.5vol%) collected with irradiation, $\lambda > 420$ nm (CysS·, marked with *).

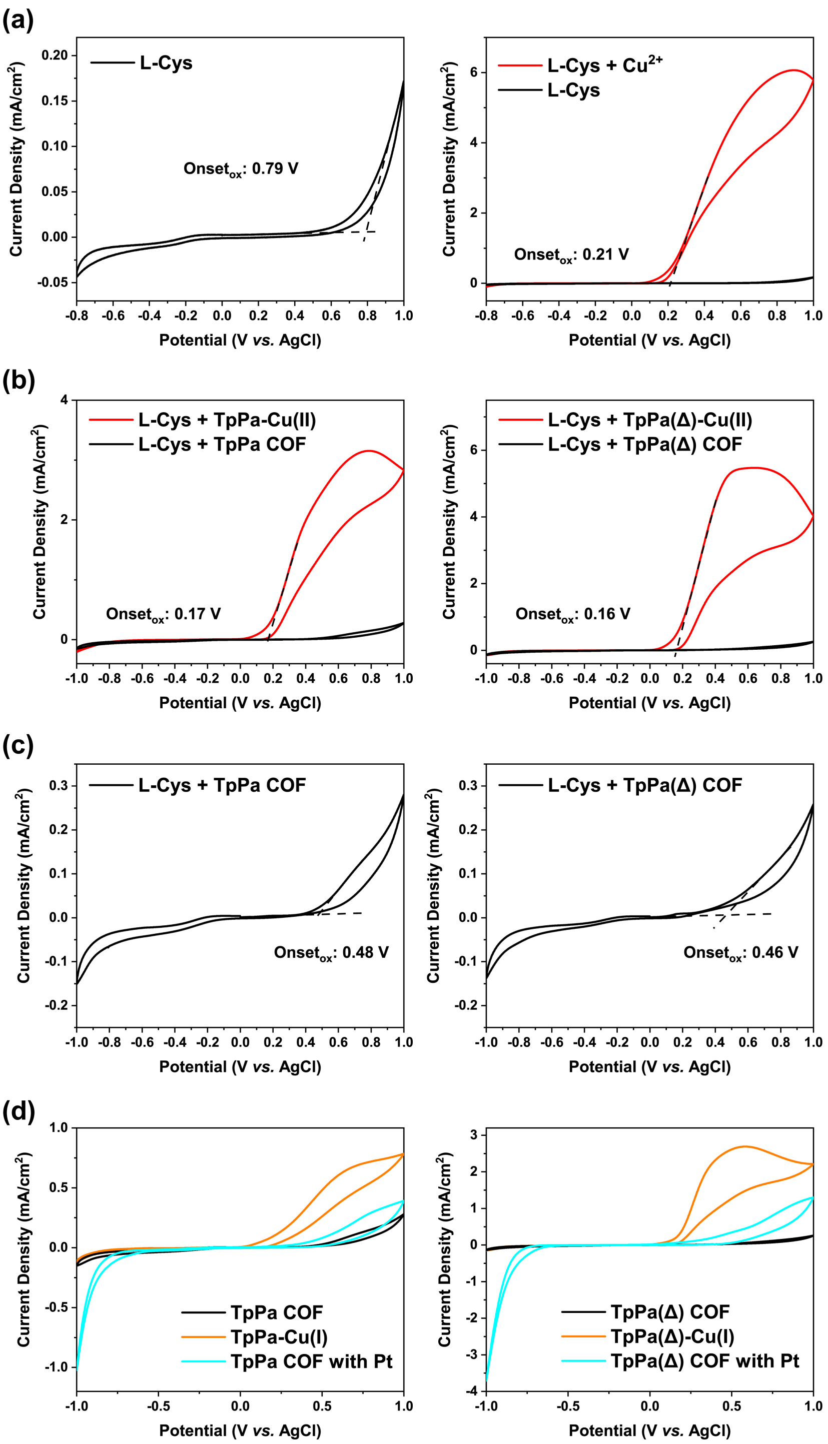

Supplementary Figure 16. Cyclic voltammogram (CV) curves.

CV curves of (a) L-cysteine (left) and L-cysteine with 0.2 mmol/L Cu^{2+} (right), (b) TpPa-COF and TpPa-Cu(II)-COF with achiral (left) and chiral (right) structures in the presence of L-cysteine, (c) achiral (left) and chiral (right) TpPa-COF in the presence of L-cysteine, and (d) TpPa-COF, TpPa-Cu(I)-COF and TpPa-COF/Pt with achiral (left) and chiral (right) structures, using the N_2-bubbled 0.2M Na_2SO_4 aqueous solution as the electrolyte at the scan rate of 100 mV s^{-1}. The concentration of L-Cysteine is 0.1 mol/L (pH=5.2).

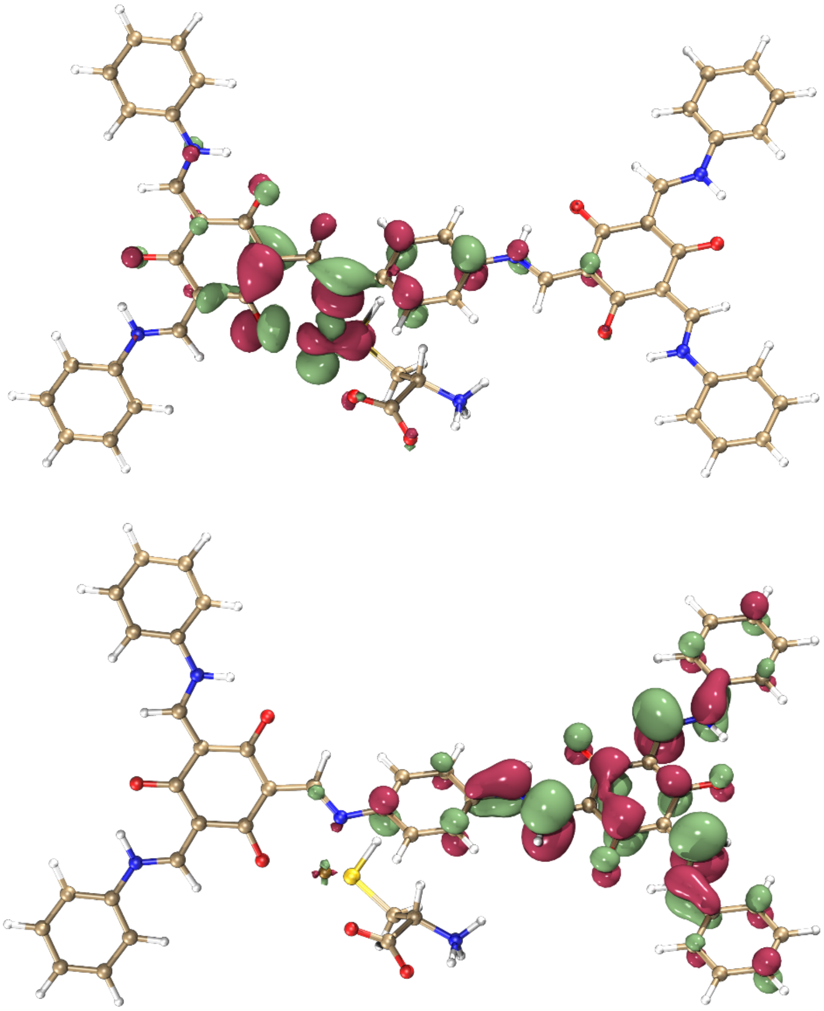

Supplementary Figure 17. DFT calculation of HOMO and LUMO.
Visualization of HOMO (up) and LUMO (down) for the TpPa-Cu(I)(16.7%) model (isovalue=0.03). C: gold, H: white, N: blue, O: red, S: yellow and Cu: brown.

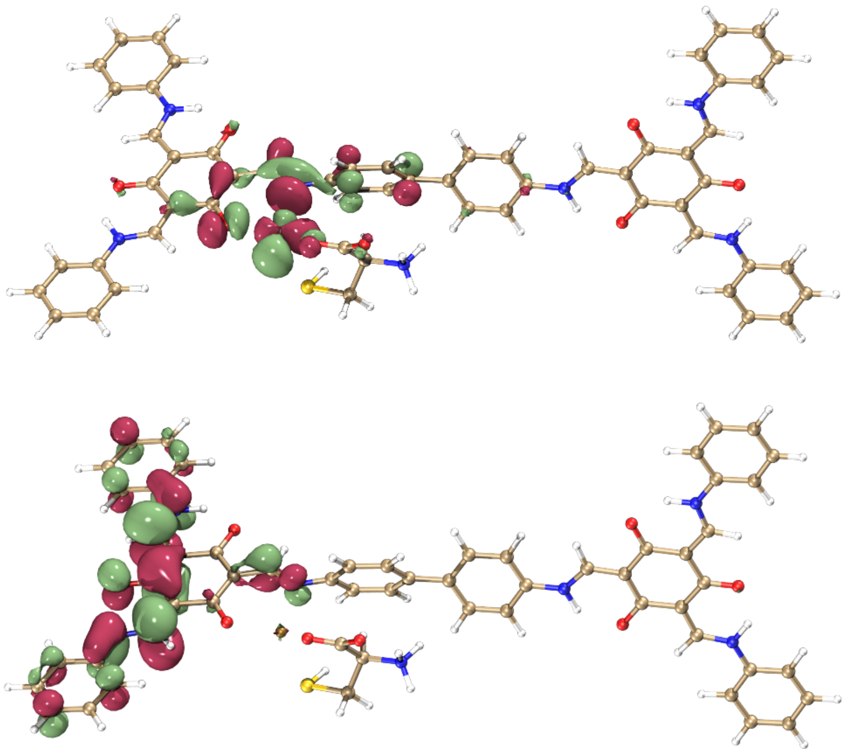

Supplementary Figure 18. DFT calculation of HOMO and LUMO+1. Visualization of HOMO (up) and LUMO+1 (down) for the TpBD-Cu(I)(16.7%) model (isovalue = 0.03). C: gold, H: white, N: blue, O: red, S: yellow and Cu: brown.

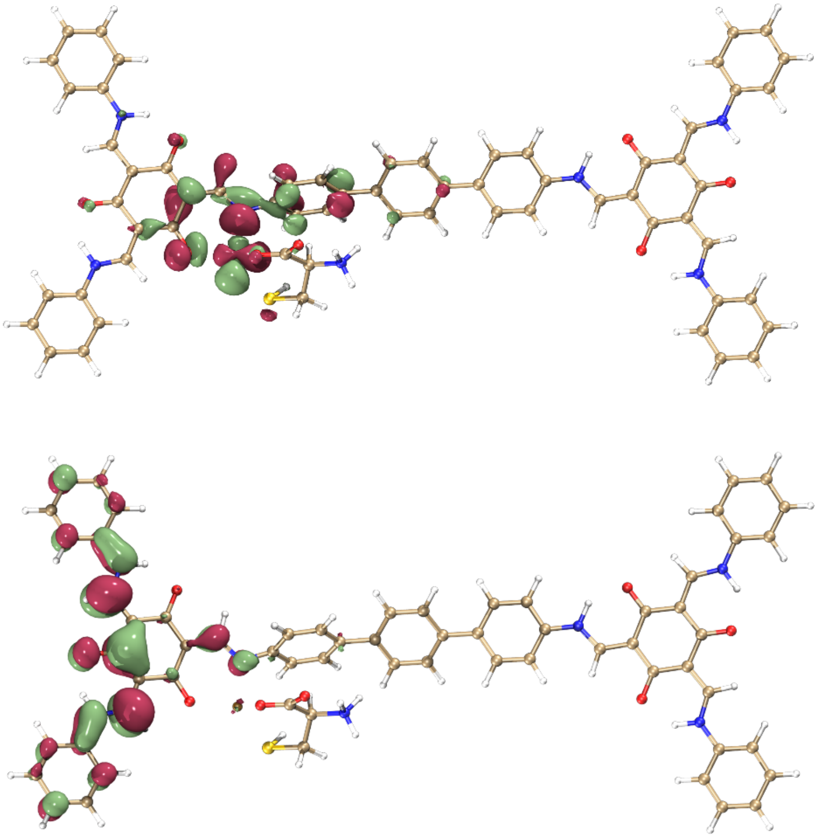

Supplementary Figure 19. DFT calculation of HOMO and LUMO+1. Visualization of HOMO (up) and LUMO+1 (down) for the TpTP-Cu(I)(16.7%) model (isovalue = 0.03). C: gold, H: white, N: blue, O: red, S: yellow and Cu: brown.

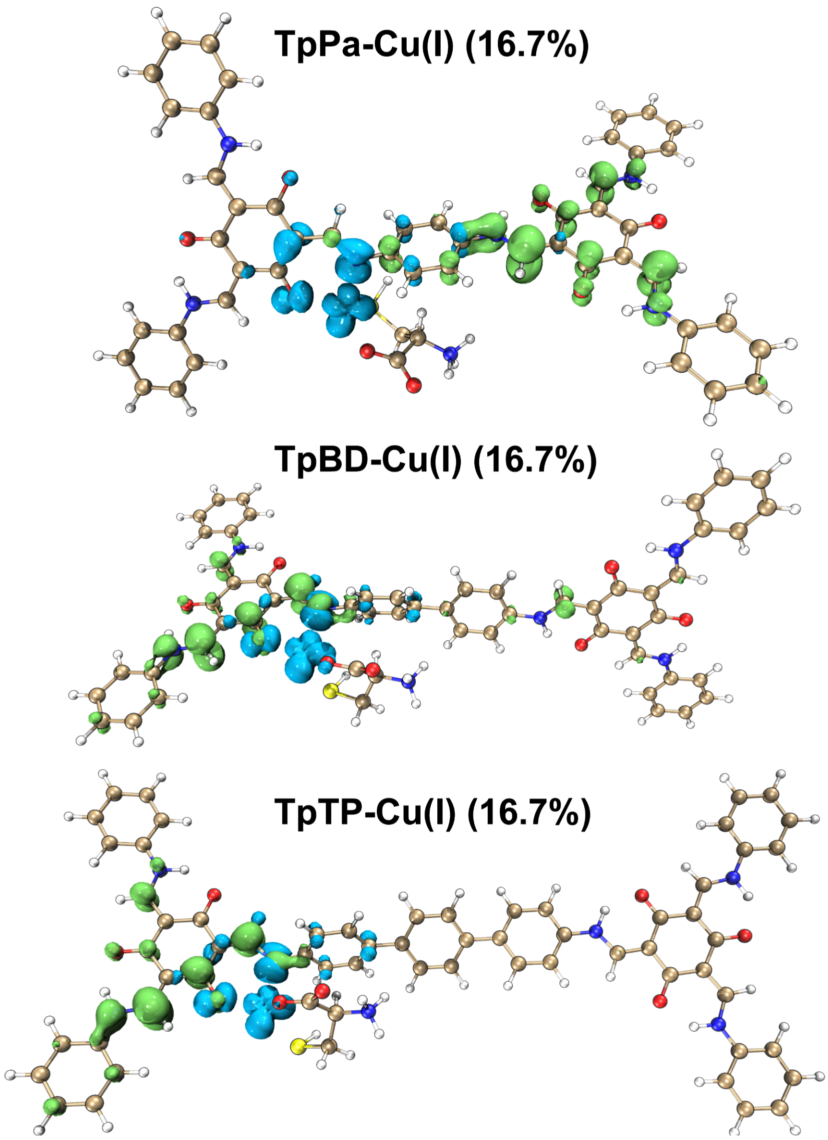

Supplementary Figure 20. Excited-state simulation.

Visualization of photo-induced electrons (green region) and holes (blue region) for the TpPa-Cu(I)(16.7%), TpBD-Cu(I)(16.7%), and TpTP-Cu(I)(16.7%), respectively (isovalue = 0.002). C: gold, H: white, N: blue, O: red, S: yellow and Cu: brown.

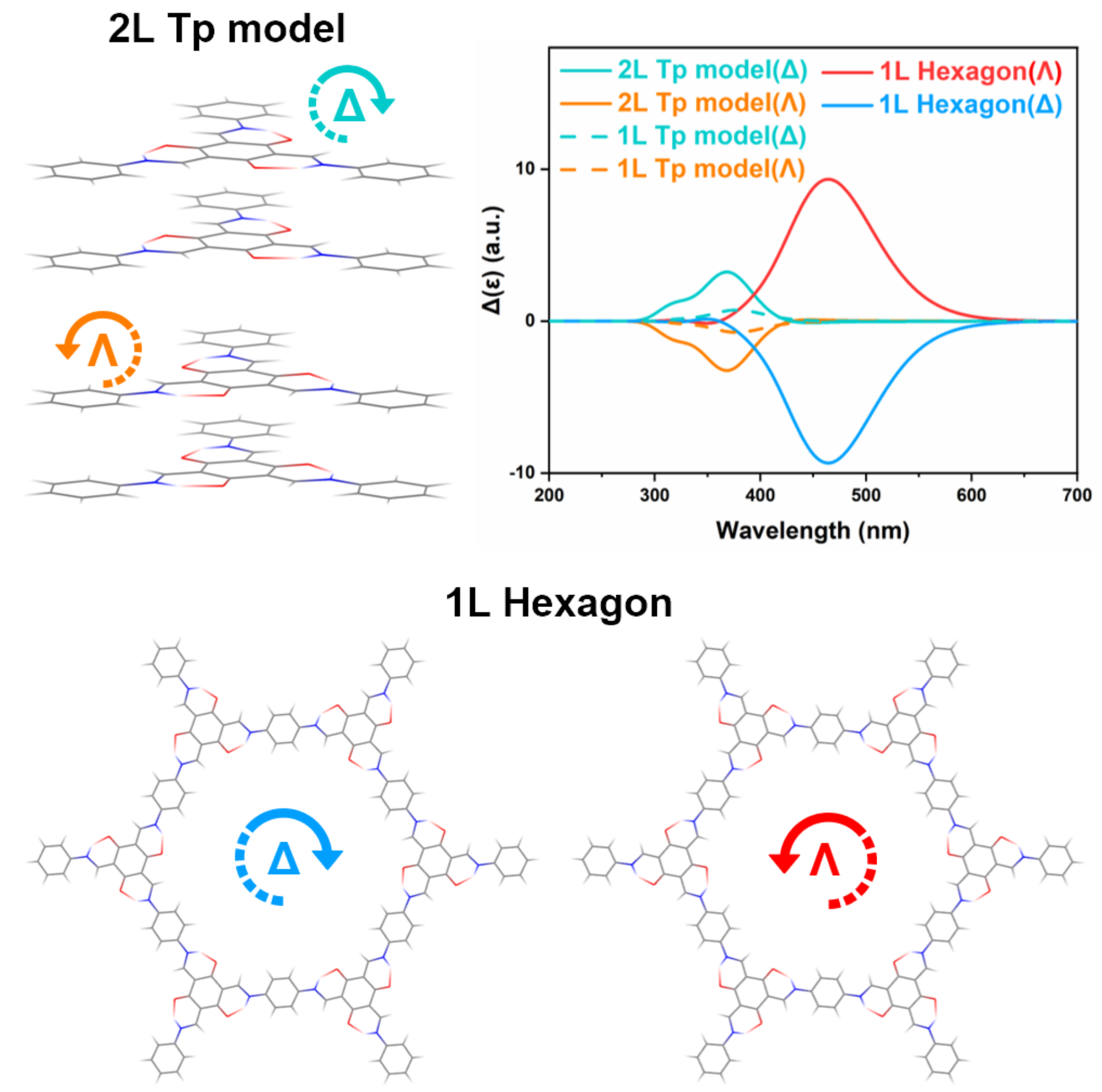

Supplementary Figure 21. Calculated electronic circular dichroism spectra. Calculated electronic circular dichroism spectra of the single-layer (1L) TpPa model, double-layer (2L) TpPa model, and single layer (1L) hexagon model. C: grey, H: white, N: blue, and O: red.

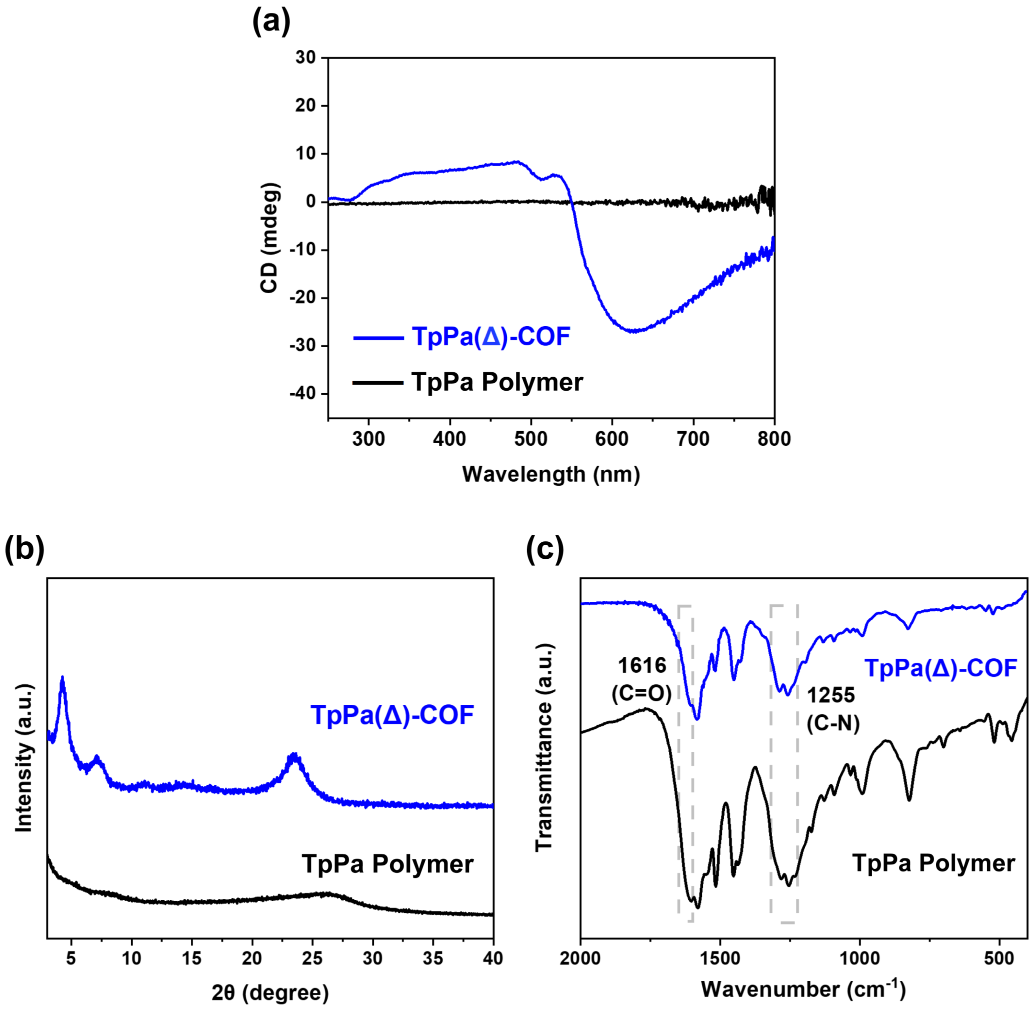

Supplementary Figure 22. Circular dichroism (CD) spectra, PXRD patterns, and FT IR spectra.

(a) CD spectra, (b) PXRD pattern, and (c) FT IR spectra of the TpPa(Δ)-COF and its corresponding amorphous polymer. The TpPa amorphous polymer was synthesized by using the chiral regulator with Tp while did not show chirality.

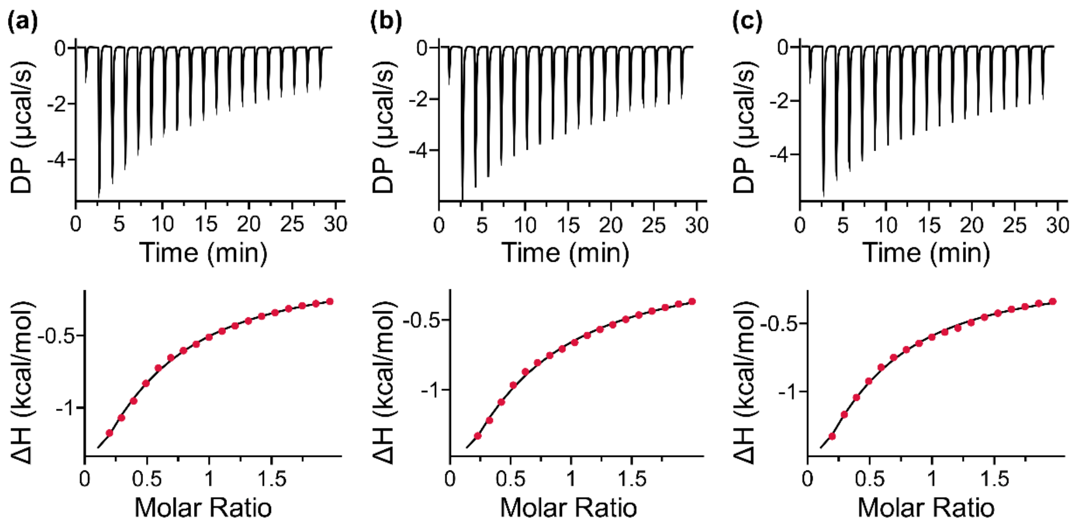

Supplementary Figure 23. Isothermal titration calorimetry thermogram. Isothermal titration calorimetry thermogram resulting from titration of (a) TpPa model compound, (b) TpPa-COF, and (c) TpPa(Δ)-COF (2 mmol/L dispersion) with 20 mmol/L Cu^{2+} aqueous solution (up), respectively, and fitting with one set of sites model (bottom).

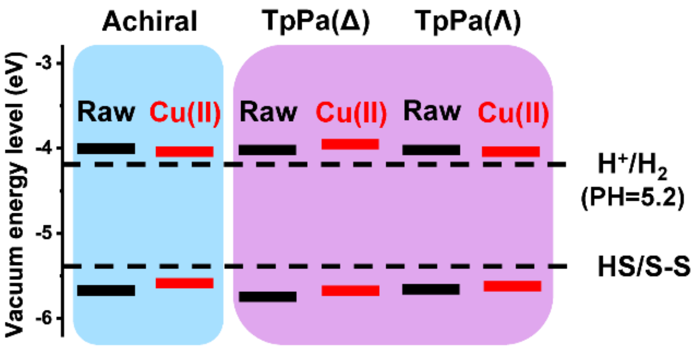

Supplementary Figure 24. Energy band structures.

Energy band structures of the achiral TpPa-COF and TpPa-Cu(II)-COF(10.76wt%) and the chiral TpPa-COFs and TpPa-Cu(II)-COFs.

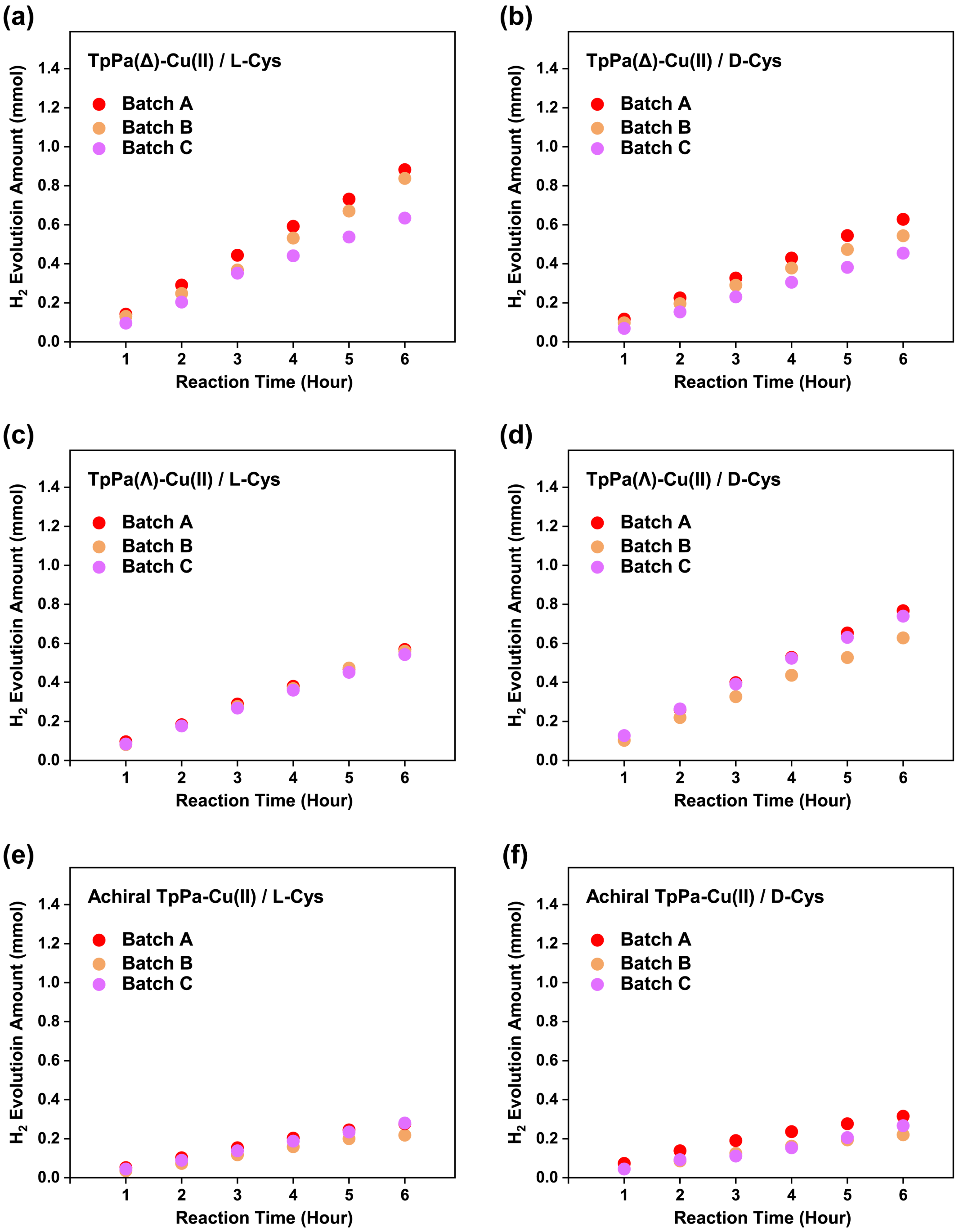

Supplementary Figure 25. Photocatalytic H_2 evolution amount *vs.* reaction time. Time courses for photocatalytic H_2 production under visible 6-h irradiation, using the various mixtures of (a) TpPa(Δ)-Cu(II)-COF/L-cysteine (L-Cys), (b) TpPa(Δ)-Cu(II)-COF/D-cysteine (D-Cys), (c) TpPa(Λ)-Cu(II)-COF/L-cysteine (L-Cys), (d) TpPa(Λ)-Cu(II)-COF/D-cysteine (D-Cys), (e) achiral TpPa-Cu(II)-COF/L-cysteine (L-Cys), and (f) achiral TpPa-Cu(II)-COF/D-cysteine (D-Cys), respectively. All the COFs used for photocatalysis were synthesized in the different batches (A, B and C) under identical conditions.

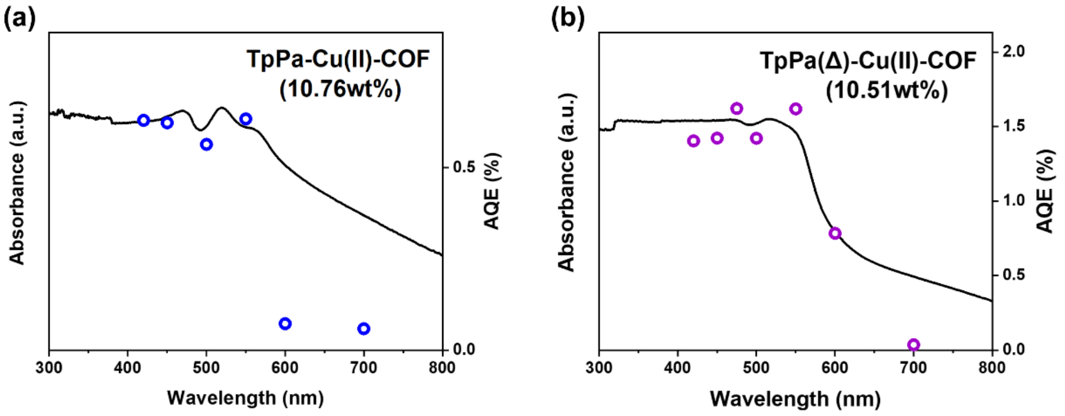

Supplementary Figure 26. Wavelength-dependent apparent quantum efficiency.

Wavelength-dependent apparent quantum efficiencies of (a) the achiral TpPa-Cu(II)-COF(10.76wt%) and (b) the chiral TpPa(Δ)-Cu(II)-COF(10.51wt%).

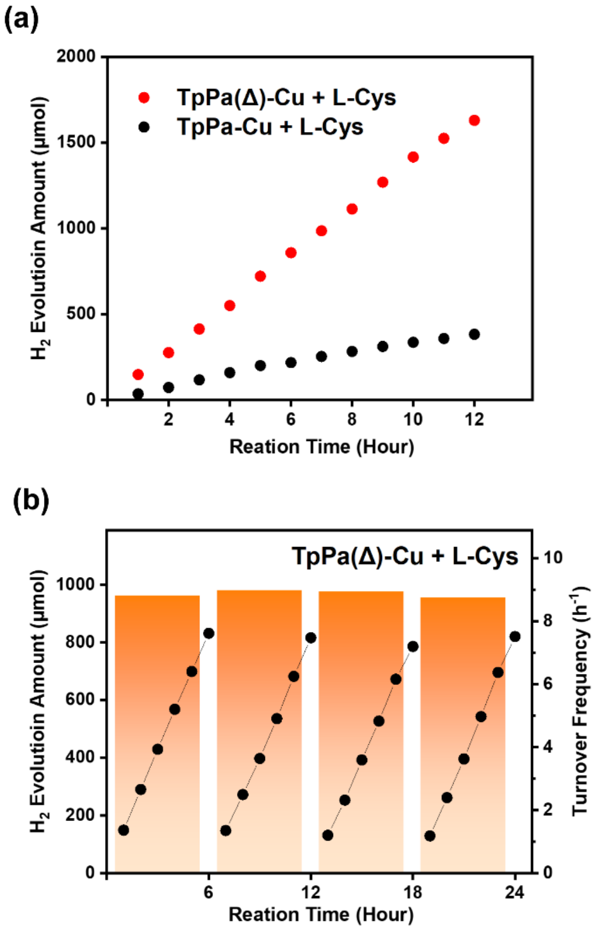

Reaction Cycles	Loaded Cu (wt%)
1	10.08
2	9.70
3	9.39
4	10.02

Supplementary Figure 27. Photocatalytic H_2 evolution amounts *vs.* reaction time and photocatalytic recycling performances.

(a) Time courses of photocatalytic H_2 evolution with TpPa-Cu(II)-COF(10.76wt%) and TpPa(Δ)-Cu(II)-COF(10.51wt%) under 12-h continuous irradiation, using L-cysteine as the SED. (b) Photocatalytic recycling performances of TpPa(Δ)-Cu(II)-COF under 24-h irradiation of visible light, including irradiation time *vs.* H_2 evolution amount (left) and 6-h averaged sacrificial turnover frequency (right). After one cycle of 6-h photocatalysis, the recycled solid was washed with 1M HCl to remove cystine that was the precipitate of cysteine oxidation reaction. Also, the remaining COF-Cu(II) complex decomposed in the strong acid. Thus, the similar content of fresh Cu(II) ions was coordinated with the recycled COF for the next photocatalytic cycle. Table in (b) shows the complexed Cu contents measured by ICP-AES.

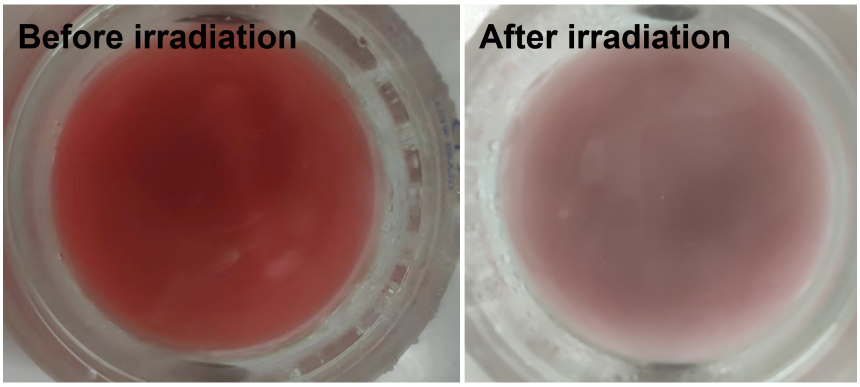

Supplementary Figure 28. Photographs of the reaction solution.
Photographs of the reaction solution before (left) and after (right) 6-h photocatalytic reaction.

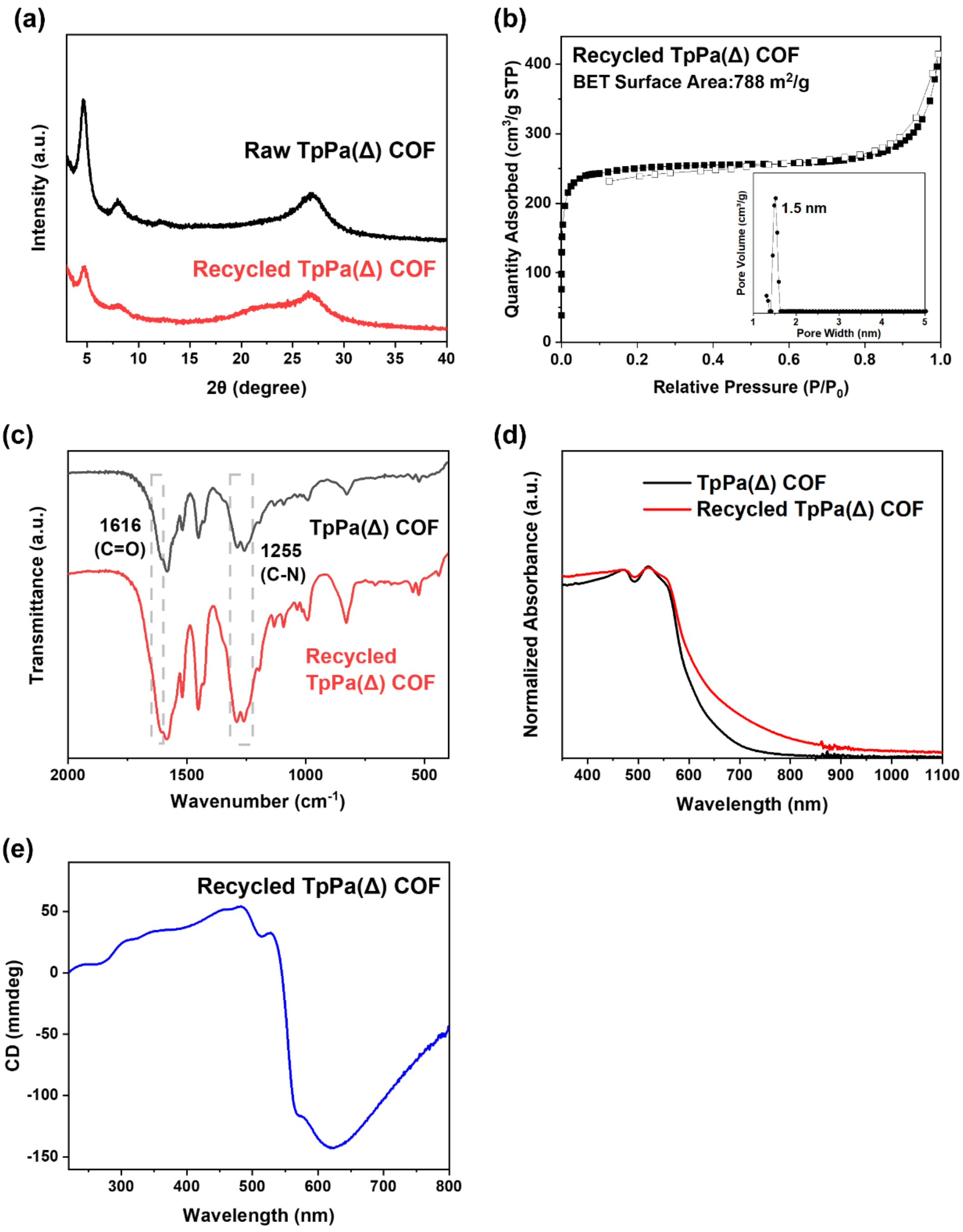

Supplementary Figure 29. PXRD pattern, N_2 sorption isotherms, pore-size distribution, FT IR spectra, UV-vis DRS spectra, and circular dichroism (CD) spectra.

(a) PXRD pattern, (b) N_2 adsorption isotherm (Inset: pore-size distribution), (c) FTIR spectra, (d) UV-vis DRS spectra and (e) CD spectra of the TpPa(Δ)-COF before and after 12-h irradiation. The recycled COF had no Cu(II) metal as the post purification of the recycled photocatalyst with 1M HCl led to the decomposition of the TpPa(Δ)-Cu(II)-COF complex. Thus, the TpPa(Δ)-COF without Cu(II) was used to compare with the remaining chiral COF.

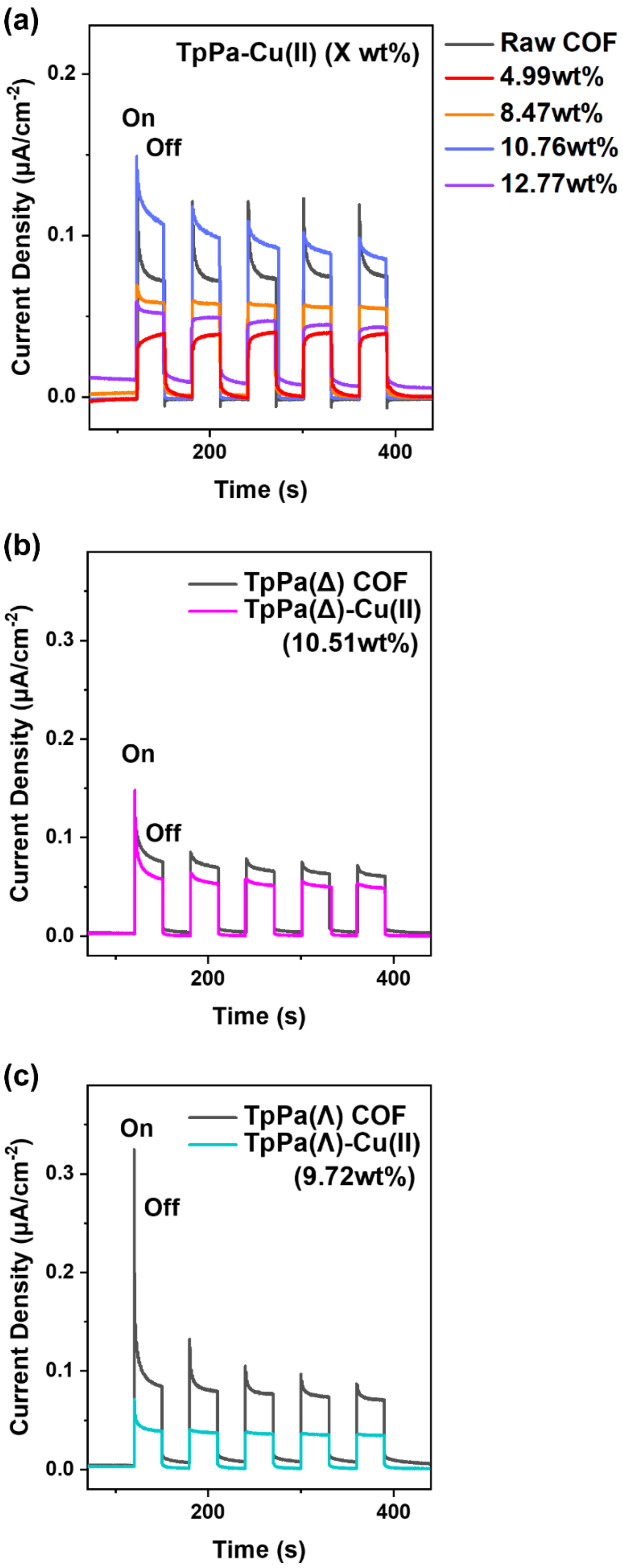

Supplementary Figure 30. Transient photocurrent response.

Transient photocurrent response of (a) TpPa-Cu(II)-COF(Xwt%) (X = 4.99, 8.47, 10.76, 12.77) and (b, c) the chiral TpPa-COF and TpPa-Cu(II)-COF.

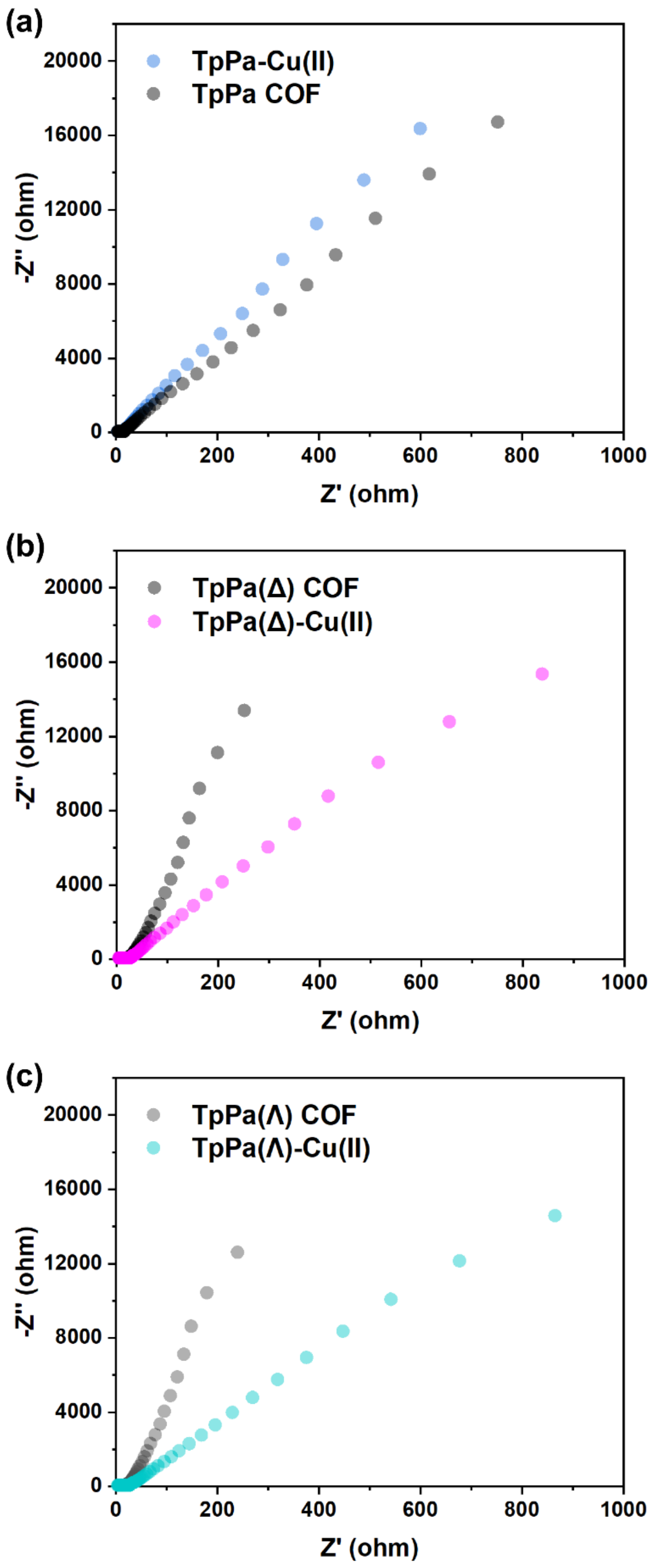

Supplementary Figure 31. Nyquist plots.

Nyquist plots of (a) the TpPa-Cu(II)-COF(10.76wt%) and (b, c) the chiral TpPa-COF and TpPa-Cu(II)-COF.

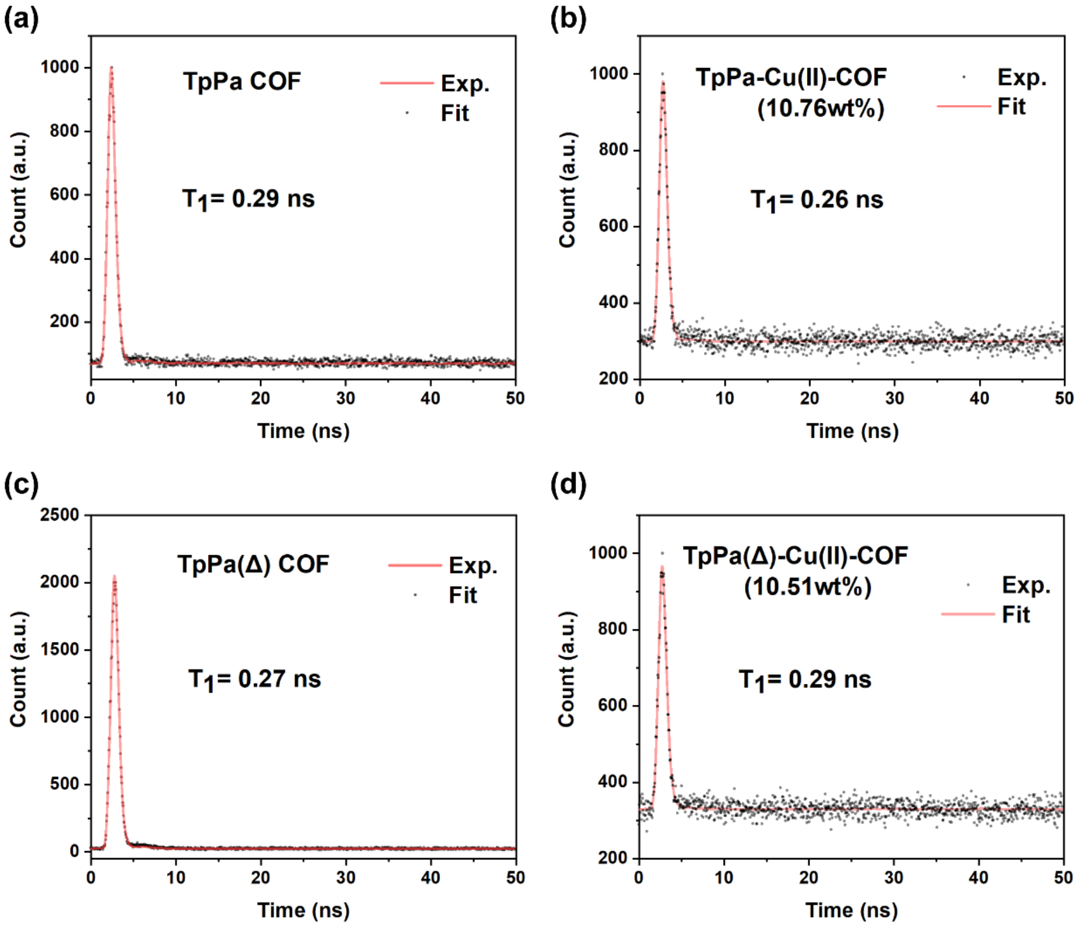

Supplementary Figure 32. Time-correlated single-photon counting measurement.

Time-correlated single-photon counting measurement of (a) TpPa-COF, (b) TpPa-Cu(II)-COF(10.76wt%), (c) TpPa(Δ)-COF, and (d) TpPa(Δ)-Cu(II)-COF(10.51wt%), respectively. The excited wavelength is 450 nm.

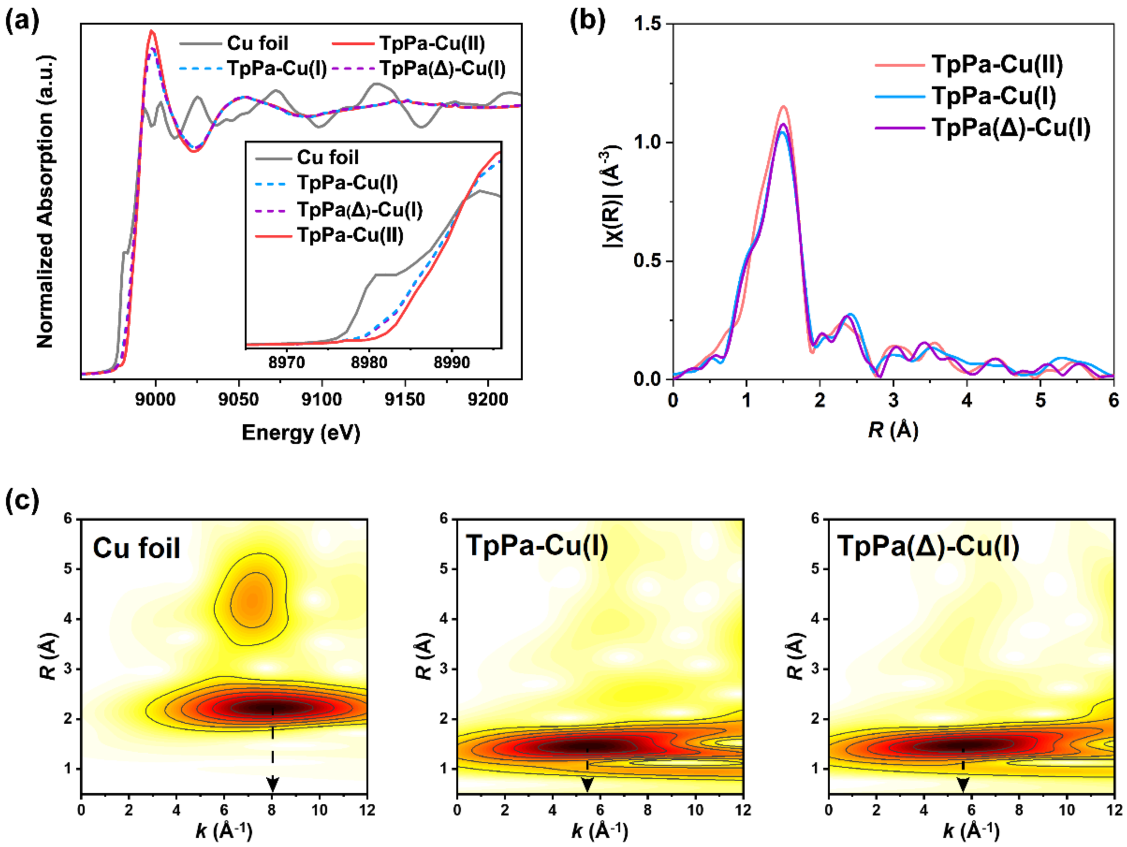

Supplementary Figure 33. EXAFS and XANES spectra, Radial distribution, and Morlet wavelet transformed X-ray absorption spectra.

(a) EXAFS and XANES (inset) spectra of the TpPa-Cu(II)-COF, TpPa-Cu(I)-COF, TpPa(Δ)-Cu(I)-COF and Cu foil. (b) Radial distribution calculated from the k^2-weighted spectrum (without phase correction) for the TpPa-Cu(II)-COF, TpPa-Cu(I)-COF, and TpPa(Δ)-Cu(I)-COF. (c) Morlet wavelet transformed X-ray absorption spectra of the Cu foil, TpPa-Cu(I)-COF, and TpPa(Δ)-Cu(I)-COF, respectively.

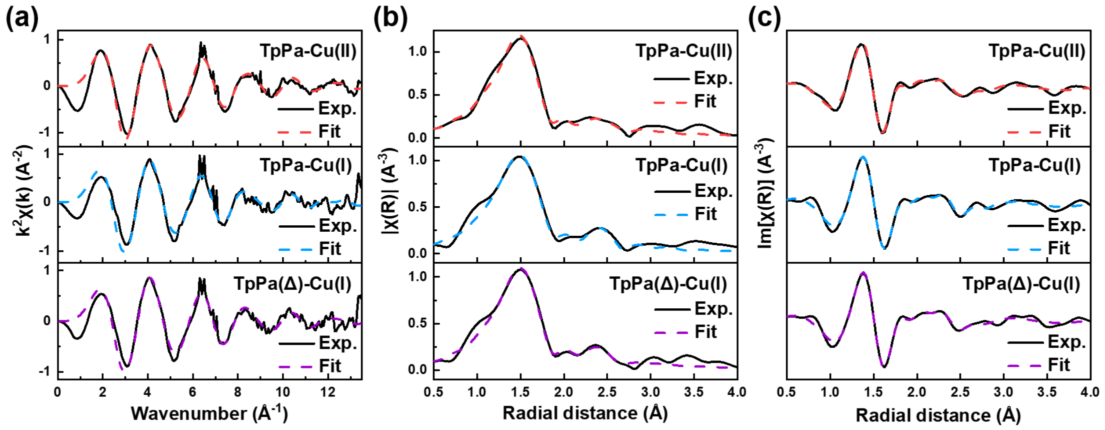

Supplementary Figure 34. EXAFS fitting spectra.

EXAFS fitting spectra for (a) the k space, (b) the r space and (c) the imaginary part of r space.

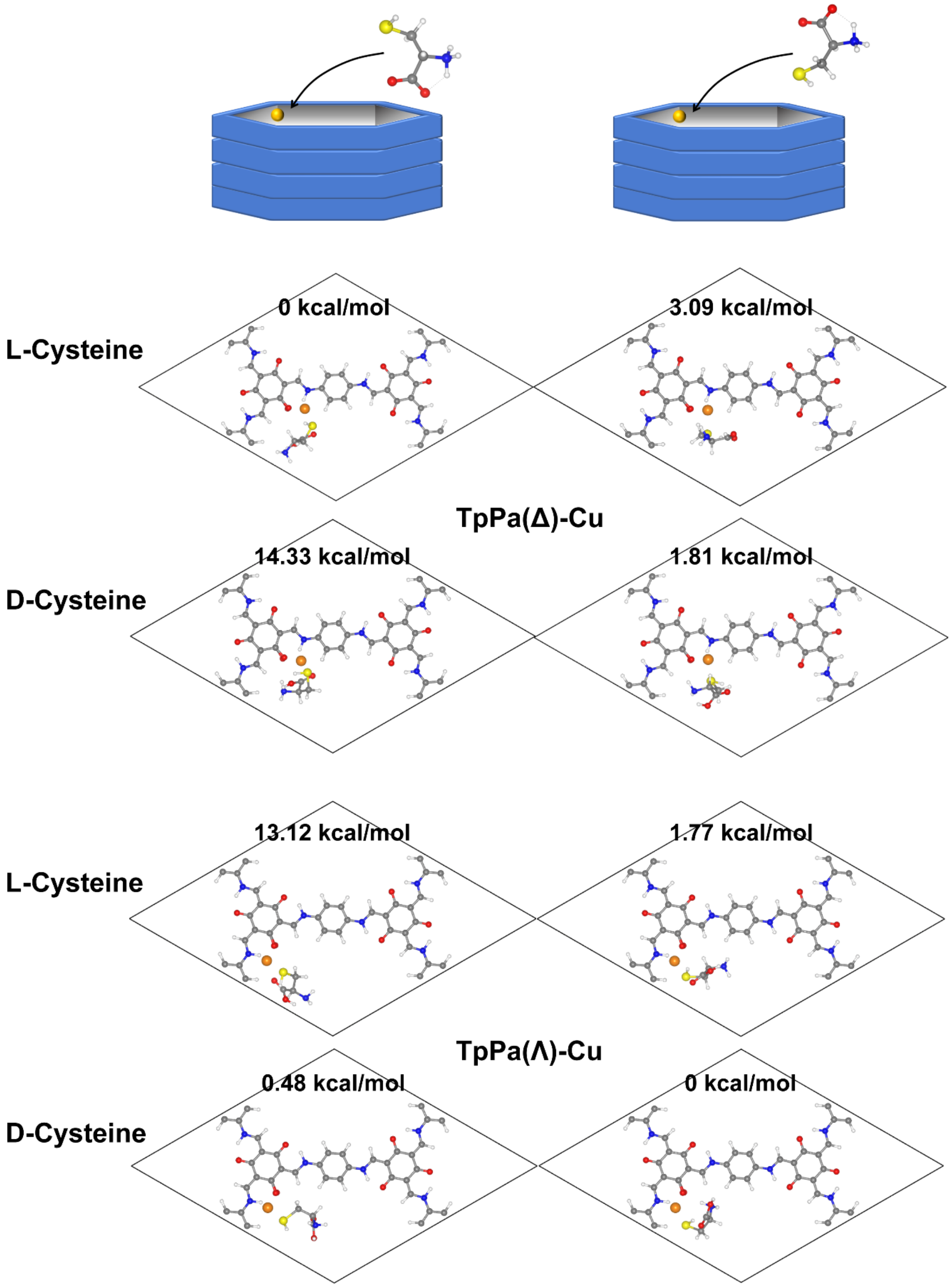

Supplementary Figure 35. Calculation of the relative Gibbs energy.
Calculation of the relative Gibbs energy for the interaction of chiral TpPa-Cu(I)-COF with L/D-cysteine in the different direction. The adopted supercell contains the two stacked COF layers. C: grey, H: white, N: blue, O: red, S: yellow and Cu: brown.

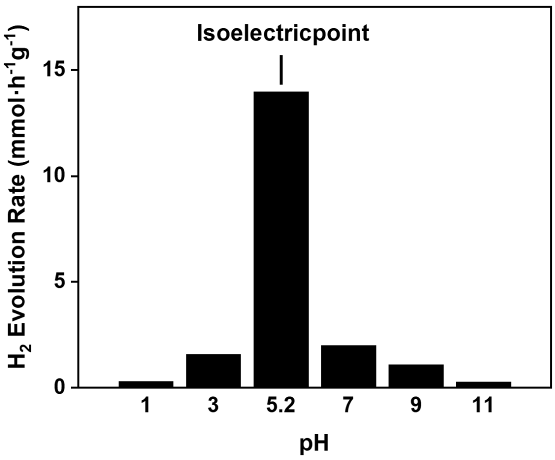

Supplementary Figure 36. pH-dependent photocatalytic HERs.

Histogram of HERs for TpPa(Δ)-Cu(II)-COF(10.39wt%) with L-cysteine under different pH values. The pH of the solution was unchanged during the photocatalytic process.

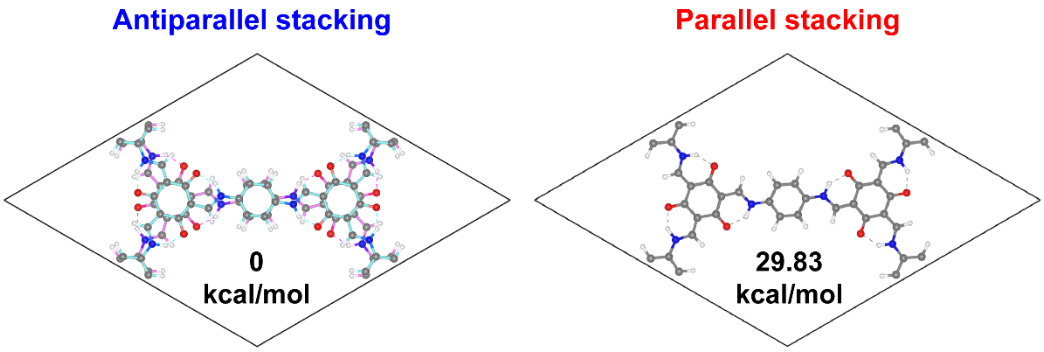

Supplementary Figure 37. Calculation of the relative Gibbs energy.

Calculation of the relative Gibbs energy for the achiral TpPa-COF (antiparallel stacking) and the chiral TpPa-COF (parallel stacking). C: grey, H: white, N: blue, and O: red.

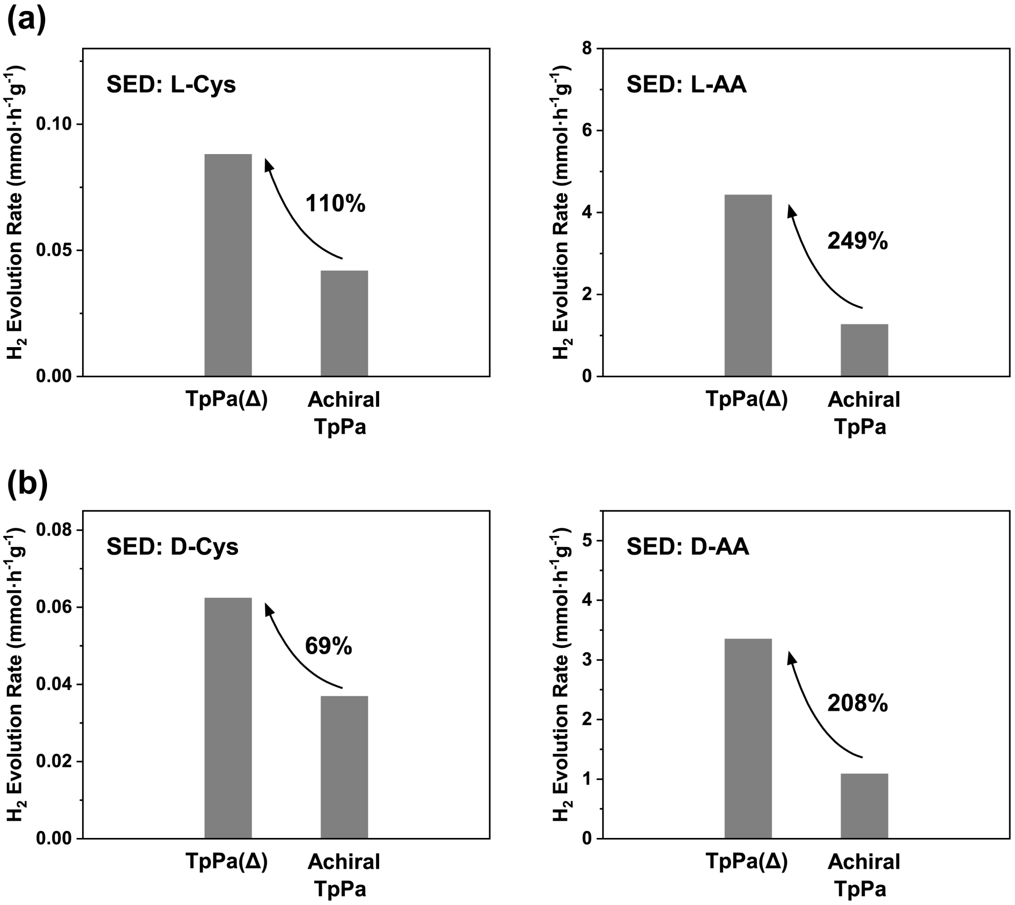

Supplementary Figure 38. Comparison of HERs.

Comparison of the HERs for the chiral and achiral TpPa-COF combined with various chiral SEDs, including (a) L-cysteine (L-Cys, left) and L-ascorbic acid (L-AA, right) and (b) D-cysteine (D-Cys, left) and D-araboascorbic acid (D-AA, right).

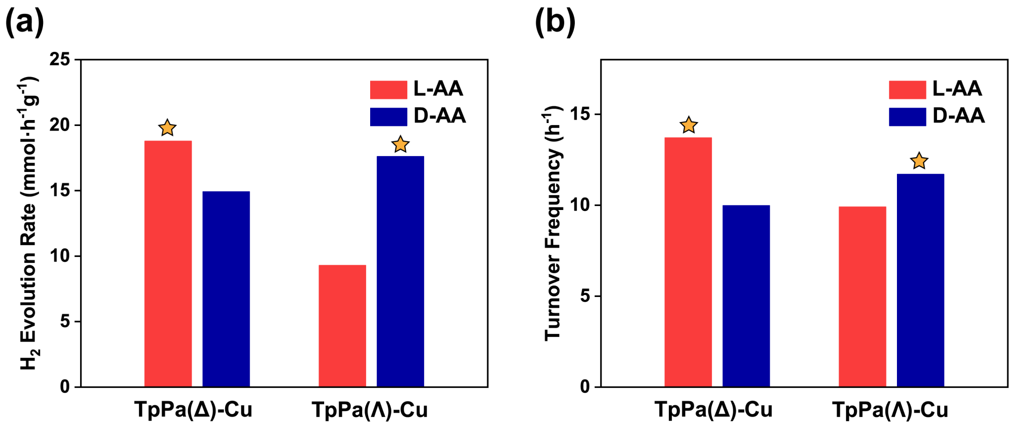

Supplementary Figure 39. Comparison of HERs and TOFs.

Comparison of (a) the HERs and (b) TOFs for the TpPa(Δ)-Cu(II)-COF(10.39wt%), TpPa(Λ)-Cu(II)-COF(9.63wt%) combined with the chiral L-ascorbic acid (L-AA) and D-araboascorbic acid (D-AA), respectively. The pH of the solution was adjusted to be 5.2 with sodium L-ascorbate or sodium D-araboascorbate. The stars on the histograms represent the different enantioselective mixtures.

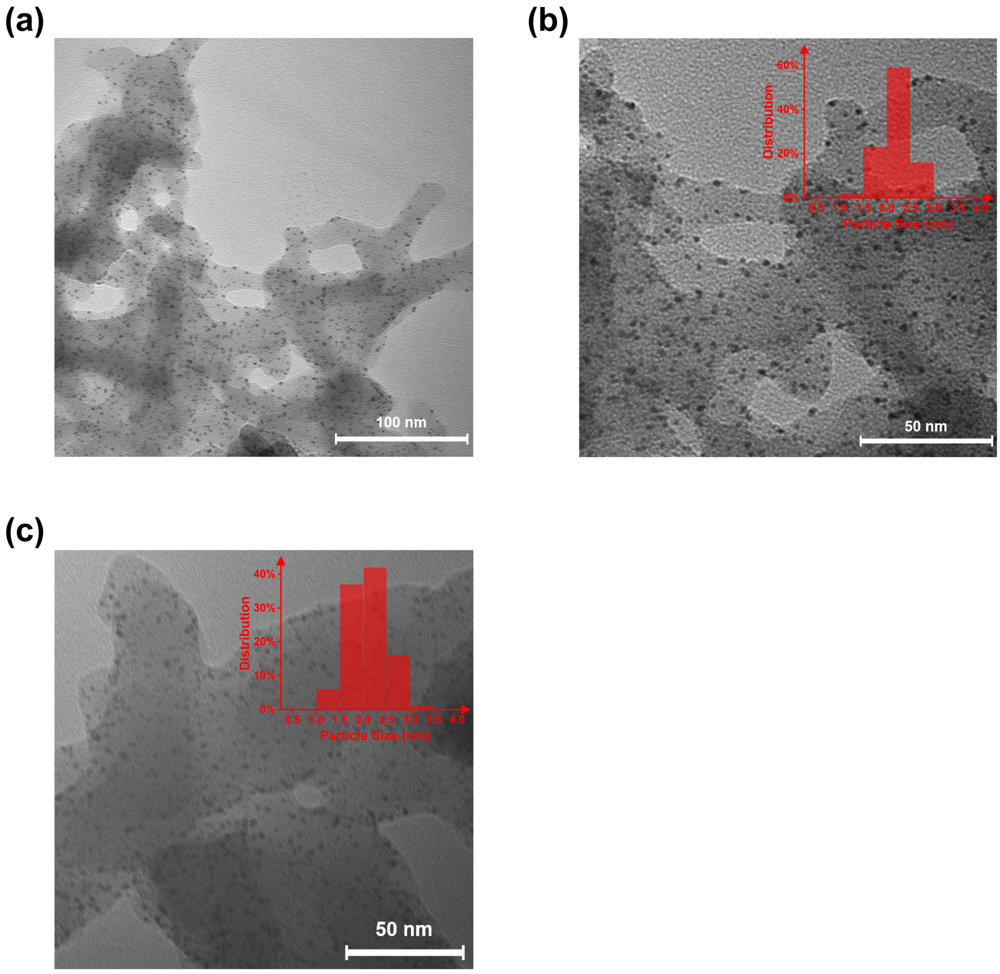

Supplementary Figure 40. TEM images.

TEM images of (a,b) the TpPa(Δ)-COF/Pt and (c) the achiral TpPa-COF/Pt. The deposited Pt amount was 2.8±0.2wt%. Insets in (b) and (c) display the statistical size distributions of the photo-deposited Pt nanoparticles.

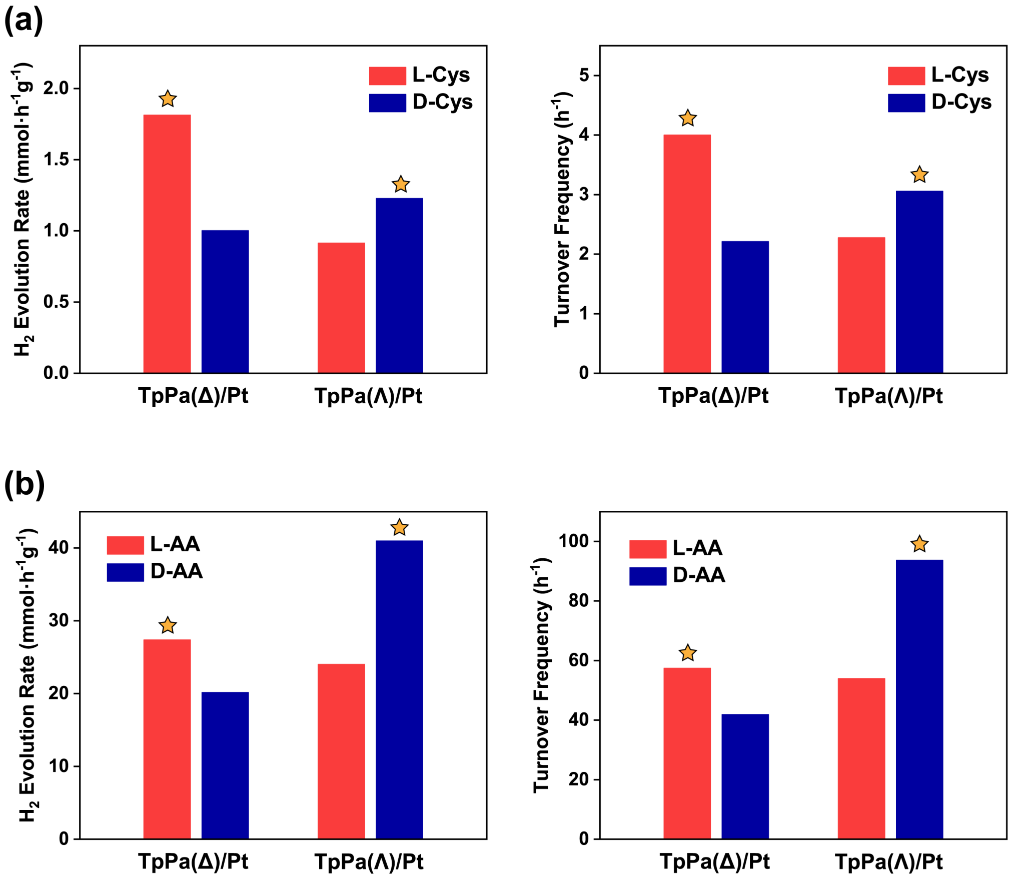

Supplementary Figure 41. Comparison of HERs and TOFs.

Comparison of the HERs and TOFs for the Pt-deposited chiral TpPa-COFs combined with (a) L-Cysteine (L-Cys) and D-Cysteine (D-Cys) and (b) L-ascorbic acid (L-AA) and D-araboascorbic acid (D-AA). The deposited Pt amount was 2.8±0.2wt% and the used chiral SEDs was 0.1M. The stars on the histograms represent the different enantiomeric mixtures.

Supplementary Table 1. The Cu contents in the samples quantified by ICP-AES.

	$Cu(OAc)_2\cdot H_2O$ (g/L)	Cu content (wt%)	*N*-salicylideneaniline subunits (mol%)
TpPa-Cu(II)-COF	2	2.90	6.3
	5	4.99	11.3
	10	8.47	20.3
	20	10.46	26.3
	30	10.76	27.6
	40	12.24	32.3
	50	12.82	34.3
	60	12.77	34.3
TpPa(Δ)-Cu(II)-COF	30	10.51	26.7
TpPa(Λ)-Cu(II)-COF	30	9.72	24.3
TpPa amorphous polymer	30	8.70	21.1
TpBD-Cu(II)-COF	5	4.29	10.0
	10	6.32	15.3
	20	10.02	26.3
	30	12.22	34.0
	40	14.27	41.7
TpTP-Cu(II)-COF	5	5.50	16.0
	10	8.99	28.0
	20	11.73	39.0
	30	13.00	44.7
	40	14.68	52.7

Supplementary Table 2. Hydrogen evolution rates (HERs) of the TpPa-Cu(II)-COF(10.76wt%) in different conditions under 6-h irradiation.

Sacrificial agent	HER (mmol $g^{-1}h^{-1}$)
10% EtOH	No H_2
10% TEOA	No H_2
0.05M Na_2SO_3	No H_2
0.1M L-Cysteine, pH = 5.2	3.64
0.05M L-Cysteine	3.59
0.01M L-Cysteine	3.18

Supplementary Table 3. Vertical excitation energy of the TpPa-Cu(I) model containing 16.7% complex, computed with the IEF-PCM implicit solvation model (water) on TD-PBE0-D3BJ/ma-def2-SVP level of theory.

State	Excitation Energy (eV)	Wavelength (nm)	Oscillator Strength	MO Transition
1	2.38	521	0.490	HOMO → LUMO (61.0%)
2	2.59	478	0.153	HOMO → LUMO+1 (50.4%)
3	2.69	461	0.042	HOMO-1 → LUMO (53.0%)
4	2.87	432	0.032	HOMO-1 → LUMO+1 (31.5%)
5	2.90	428	0.000	HOMO → LUMO+4 (50.2%)

Supplementary Table 4. Vertical excitation energy of the TpBD-Cu(I) model containing16.7% complex, computed with the IEF-PCM implicit solvation model (water) on TD-PBE0-D3BJ/ma-def2-SVP level of theory.

**State**	**Excitation Energy (eV)**	**Wavelength (nm)**	**Oscillator Strength**	**MO Transition**
1	2.47	502	0.283	HOMO → LUMO+1 (53.3%)
2	2.63	472	0.272	HOMO → LUMO (61.1%)
3	2.74	453	0.158	HOMO-1 → LUMO+1 (49.9%)
4	2.87	432	0.030	HOMO-1 → LUMO (56.6%)
5	2.90	427	0.002	HOMO → LUMO+4 (73.9%)

Supplementary Table 5. Vertical excitation energy of the TpTP-Cu(I) model containing 16.7% complex, computed with the IEF-PCM implicit solvation model (water) on TD-PBE0-D3BJ/ma-def2-SVP level of theory.

**State**	**Excitation Energy (eV)**	**Wavelength (nm)**	**Oscillator Strength**	**MO Transition**
1	2.59	479	0.300	HOMO → LUMO+1 (70.0%)
2	2.78	447	0.478	HOMO → LUMO (76.5%)
3	2.89	429	0.159	HOMO-1 → LUMO+1 (70.4%)
4	3.02	411	0.008	HOMO → LUMO+4 (72.5%)
5	3.06	406	0.632	HOMO-1 → LUMO (48.8%)

Supplementary Table 6. The transferred photo-induced electrons calculated by IFCT method.

**Computational model**	**Cu(I) complex →Tp**	**Complexed ligand[a] →Tp**	**Complexed Cu(I)→Tp**
TpPa-Cu(I)(16.7%) model	0.49e	0.20e	0.29e
TpBD-Cu(I)(16.7%) model	0.11e	0.03e	0.08e
TpTP-Cu(I)(16.7%) model	0.03e	0.01e	0.02e

[a]The ligand for the coordination with Cu(I) is *N*-salicylideneaniline subunit at the knot of COF framework.

Supplementary Table 7. The fitting results of isothermal titration calorimetry (Supplementary Fig. 23) using one set of sites model. KD presents the dissociation constant.

	**N (Sites)**	**KD (M)**	**ΔG (kcal mol^{-1})**
TpPa model	0.255	2.04×10^{-3}	-3.67
TpPa-COF	0.252	2.53×10^{-3}	-3.54
TpPa(Δ)-COF	0.250	1.88×10^{-3}	-3.72

Supplementary Table 8. Comparison of photocatalytic hydrogen evolution performances with the different metal photocatalysts.

Photocatalysts/Cocatalyst	Irradiation	Sacrificial agent	Solvent	H_2 evolution rate	Ref.
**TpPa(Δ)-Cu(II)-COF**	**>420nm (200mW cm^{-2})**	**L-Cysteine**	**H_2O**	**14.7 mmol $g^{-1}h^{-1}$**	**This work**
N1-COF/Co-1	AM1.5	TEOA	MeCN/H_2O	100 μmol $g^{-1}h^{-1}$	[15]
N2-COF/Co-1	AM1.5	TEOA	MeCN/H_2O	782 μmol $g^{-1}h^{-1}$	[15]
TpDTz COF/NiME	AM1.5	TEOA	H_2O	941μmol $g^{-1}h^{-1}$	[16]
TpPa-1 COF/MoS_2	>420nm	Ascorbic acid	H_2O	5.6 mmol $g^{-1}h^{-1}$	[17]
NUS-55/[Co(bpy)$_3$]Cl_2	>420nm	TEA	EtOH/H_2O	2.5 mmol $g^{-1}h^{-1}$	[18]
Cobaloxime-COF/Co(II)	AM1.5	TEOA	MeCN/H_2O	163 μmol $g^{-1}h^{-1}$	[19]
Ti_3-BPDC-Ru	>400nm	BIH	MeCN	61 μmol $g^{-1}h^{-1}$	[20]
Ti_3-BPDC-Lr	>400nm	BIH	MeCN	925 μmol $g^{-1}h^{-1}$	[20]
MIL-125-NH_2/Co(II)	>380nm	TEOA	MeCN	553 μmol $g^{-1}h^{-1}$	[21]
NH_2-MIL125/Co(III)	>408nm	TEA	MeCN	380 μmol $g^{-1}h^{-1}$	[22]
MOF-5/Ni	>420nm	TEOA	H_2O	3.0 mmol $g^{-1}h^{-1}$	[23]
Ni_4P_2@MOF	>400nm	MeOH	H_2O	4.4 mmol $g^{-1}h^{-1}$	[24]

$C_3N_4/CoL_2(SCN)_2$	>420nm	TEOA	H_2O	272 μmol $g^{-1}h^{-1}$	[25]
$[Lr(ppy)_2(dtbbpy)][PF_6]/Ni_4P_2$	455nm (20mW)	TEOA	H_2O	40 μmol/day	[26]
3DOM-$BiVO_4$	200mW cm^{-2}	Na_2SO_3	H_2O	260 μmol $g^{-1}h^{-1}$	[27]
Ni_4P_2-CQD@CdS	420nm (100mW cm^{-2})		H_2O	145 μmol $g^{-1}h^{-1}$	[28]
TC/N-ZIS/$Ti_3C_2T_x$	300W Xe lamp	TEOA	H_2O	148 μmol $g^{-1}h^{-1}$	[29]
MnO_x/CdS/CoP	AM1.5	Na_2S/Na_2SO_3	H_2O	238 μmol $g^{-1}h^{-1}$	[30]
TiO_2/MoS_2(E)	300W Xe lamp	EtOH	H_2O	4.3 mmol $g^{-1}h^{-1}$	[31]
$Cu_2(OH)_2CO_3/TiO_2$	300W Xe lamp	MeOH	H_2O	6.7 mmol $g^{-1}h^{-1}$	[32]
$ZnSe/Ni(BF_4)_2$	AM1.5	Ascorbic acid	H_2O	54.3 mmol $g^{-1}h^{-1}$	[33]
LnP/ZnS/Ni	AM1.5	Ascorbic acid	H_2O	70 mmol $g^{-1}h^{-1}$	[34]
TpPa-COF/Pt-PVP	>420nm	Ascorbic acid	H_2O	8.4 mmol $g^{-1}h^{-1}$	[35]
TpPa-COF/Pt single-atom	>420nm (265mW cm^{-2})	Sodium ascorbate	H_2O(PBS)	719 μmol $g^{-1}h^{-1}$	[36]

Supplementary Table 9. The EXAFS fitting results including coordination number (N), interatomic distance (R_{eff}), interatomic distance error (Δr), Debye-Waller factor (σ^2), edge energy shift (ΔE_0), and R factor.

**Path**	**R_{eff}(Å)**	**ΔE_0**	**N**	**Δr(Å)**	**σ^2**	**R(%)**
TpPa-Cu(II)_Cu-O	1.845	1.228	4.500	0.082	0.006	1.8
TpPa-Cu(II)_Cu-C	2.761	1.228	1.126	0.104	0.005	1.8
TpPa-Cu(I)_Cu-O	1.980	-1.812	4.636	-0.038	0.007	1.3
TpPa-Cu(I)_Cu-C	2.767	-1.812	0.702	0.085	0.00007	1.3
TpPa(Δ)-Cu(I)_Cu-O	1.950	-1.499	4.224	-0.011	0.006	0.9
TpPa(Δ)-Cu(I)_Cu-C	2.797	-1.499	0.486	0.049	0.003	0.9

Supplementary References

1. R. Wang, W. Kong, T. Zhou, et al. Organobase modulated synthesis of high-quality β-ketoenamine-linked covalent organic frameworks. *Chem. Commun.* **57**, 331-334 (2021).
2. B. Ravel and M. Newville, ATHENA, ARTEMIS, HEPHAESTUS: data analysis for X-ray absorption spectroscopy using IFEFFIT, *Journal of Synchrotron Radiation* **12**, 537–541 (2005).
3. (a) Funke, H.; Scheinost, A. C.; Chukalina, M. Wavelet analysis of extended X-ray absorption fine structure data. *Physical Review B* **71**, 094110 (2005); (b) Funke, H. Chukalina, M.; Scheinost, A. C. A new FEFF-based wavelet for EXAFS data analysis. *Journal of Synchrotron Radiation* 14, 426-432 (2007).
4. M. J. Frisch, et al. *Gaussian16 Revision A.03* (Gaussian, Inc., 2016).
5. S. Grimme, S. Ehrlich, L. Goerigk. Effect of the damping function in dispersion corrected density functional theory. *J. Comput. Chem.* **32**, 1456-1465 (2011).
6. Zheng, J., Xu, X. & Truhlar, D.G. Minimally augmented Karlsruhe basis sets. *Theor. Chem. Acc.* **128**, 295–305 (2011).
7. Tian Lu, Feiwu Chen. Multiwfn: A multifunctional wavefunction analyzer. *J. Comput. Chem.* **33**, 580-592 (2012).
8. W. Humphrey, A. Dalke, K. Schulten. VMD: Visual molecular dynamics. *J. Mol. Graph. Model.* **14**, 33-38 (1996).
9. R. Parsons. The rate of electrolytic hydrogen evolution and the heat of adsorption of hydrogen. *Trans. Faraday Soc.* **54**, 1053–1063 (1958).
10. Kuehne,Thomas D., Iannuzzi,Marcella, Del Ben,Mauro, et al. CP2K: An electronic structure and molecular dynamics software package - Quickstep: Efficient and accurate electronic structure calculations. *J. Chem. Phys.* **152**, 194103 (2020).
11. K. Momma, F. Izumi. VESTA 3 for three-dimensional visualization of crystal, volumetric and morphology data. *J. Appl. Crystallogr.* **44**, 1272-1276 (2011).
12. S. Kandambeth, A. Mallick, B. Lukose, et al. Construction of crystalline 2D covalent organic frameworks with remarkable chemical (acid/base) stability via a combined reversible and irreversible route. *J. Am. Chem. Soc.* **134**, 19524-19527

(2012).

13. Tian Lu, Qinxue Chen. Shermo: A general code for calculating molecular thermodynamic properties. *Comput. Theor. Chem.* **1200**, 113249 (2021).
14. J. K. Nørskov, J. Rossmeisl, A. Logadottir, et al. Origin of the Overpotential for Oxygen Reduction at a Fuel-Cell Cathode. *J. Phys. Chem. B* **108**, 17886-17892 (2004).
15. Tanmay Banerjee, et al. Single-site photocatalytic H_2 evolution from covalent organic frameworks with molecular Cobaloxime Co-catalysts. *J. Am. Chem. Soc.* **139**, 16228-16234 (2017).
16. Bishnu P. Biswal, et al. Sustained solar H_2 evolution from a Thiazolo[5,4-d]thiazole-bridged covalent organic framework and Nickel-Thiolate cluster in water. *J. Am. Chem. Soc.* **141**, 11082-11092 (2019).
17. Meng-Yao Gao, et al. Boosting visible-light-driven hydrogen evolution of covalent organic frameworks through compositing with MoS_2: a promising candidate for noble-metal-free photocatalysts. *J. Mater. Chem. A* **7**, 20193-20200 (2019).
18. Jian W., et al. Cobalt-containing covalent organic frameworks for visible light-driven hydrogen evolution. *Sci. China Chem.* **63**, 192–197 (2020).
19. Kerstin Gottschling, et al. Rational design of covalent Cobaloxime–covalent organic framework hybrids for enhanced photocatalytic hydrogen evolution. *J. Am. Chem. Soc.* **142**, 12146-12156 (2020).
20. Yang S., et al. Titanium hydroxide secondary building units in metal–organic frameworks catalyze hydrogen evolution under visible light. *J. Am. Chem. Soc.* **141**, 12219−12223 (2019).
21. Zhe Li, et al. Encapsulating a Co(II) molecular photocatalyst in metal–organic framework for visible-light-driven H_2 production: boosting catalytic efficiency via spatial charge separation. *ACS Catal.* **6** (8), 5359-5365 (2016).
22. M. A. Nasalevich, et al. Co@NH_2-MIL-125(Ti): cobaloxime-derived metal–organic framework-based composite for light-driven H_2 production. *Energ. Environ. Sci.* **8** (1), 364-375 (2015).
23. Wenlong Zhen, et al. Small-sized Ni(1 1 1) particles in metal-organic frameworks

with low over-potential for visible photocatalytic hydrogen generation. *Appl. Catal. B* **190**, 12-25 (2016).

24. Xiangjian Kong, et al. Hierarchical integration of photosensitizing metal–organic frameworks and Nickel-containing polyoxometalates for efficient visible-light-driven hydrogen evolution. *Angew. Chem. Int. Ed.* **55**, 6411-6416 (2016).
25. Junshuai Zhang, et al. Superior photocatalytic generation of H_2 in water medium through grafting a Cobalt molecule co-catalyst from carbon nitride nanosheets. *ChemCatChem* **11**, 2657–2666 (2019).
26. Hongjin Lv, et al. A noble-metal-free, tetra-nickel polyoxotungstate catalyst for efficient photocatalytic hydrogen evolution. *J. Am. Chem. Soc.* **136** (40), 14015–14018 (2014).
27. Hao Wu, et al. Unveiling carrier dynamics in periodic porous $BiVO_4$ photocatalyst for enhanced solar water splitting. *ACS Energy Lett.* **6** (10), 3400–3407 (2021).
28. Yinjuan Dong, et al. Carbon quantum dots enriching molecular nickel polyoxometalate over CdS semiconductor for photocatalytic water splitting. *Appl. Catal. B* **293** (15), 120214 (2021).
29. Tongming Su, et al. Sulfur vacancy and $Ti_3C_2T_x$ cocatalyst synergistically boosting interfacial charge transfer in 2D/2D $Ti_3C_2T_x/ZnIn_2S_4$ heterostructure for enhanced photocatalytic hydrogen evolution. *Adv. Sci.* **9**, 2103715 (2022).
30. Xing, M. Y., et al. Spatially separated CdS shells exposed with reduction surfaces for enhancing photocatalytic hydrogen evolution. *Adv. Funct. Mater.* **27**, 1702624 (2017).
31. Haiyong He, et al. MoS_2/TiO_2 edge-on heterostructure for efficient photocatalytic hydrogen evolution. *Adv. Energy Mater.* **6**, 1600464 (2016).
32. Zhikang He, et al. $Cu_2(OH)_2CO_3$ clusters: novel noble-metal-free cocatalysts for efficient photocatalytic hydrogen production from water splitting. *Appl. Catal., B* **205**, 104-111 (2017).
33. M. F. Kuehnel, et al. ZnSe nanorods as visible-light absorbers for photocatalytic and photoelectrochemical H_2 evolution in water. *Angew. Chem. Int. Ed.* **58**, 5059-5063 (2019).

34. Shan Yu, et al. Efficient photocatalytic hydrogen evolution with ligand engineered all-inorganic InP and InP/ZnS colloidal quantum dots. *Nat. Commun.* **9**, 4009 (2018).

35. J. Ming, et al. Hot π-Electron Tunneling of Metal–Insulator–COF Nanostructures for Efficient Hydrogen Production. *Angew. Chem. Int. Ed.* **58**, 18290-18294 (2019).

36. P. Dong, et al. Platinum Single Atoms Anchored on a Covalent Organic Framework: Boosting Active Sites for Photocatalytic Hydrogen Evolution. *ACS Catal.* **11**, 13266–13279 (2021).
